# Genome-Wide Association Studies Reveal Susceptibility Loci for Noninfectious Claw Lesions in Holstein Dairy Cattle

**DOI:** 10.3389/fgene.2021.657375

**Published:** 2021-05-28

**Authors:** Ellen Lai, Alexa L. Danner, Thomas R. Famula, Anita M. Oberbauer

**Affiliations:** Animal Science Department, University of California, Davis, Davis, CA, United States

**Keywords:** sole ulcer, pododermatitis circumscripta, white line disease, lameness, genome-wide association study, random forest, Bayesian regression, dairy cattle

## Abstract

Sole ulcers (SUs) and white line disease (WLD) are two common noninfectious claw lesions (NICL) that arise due to a compromised horn production and are frequent causes of lameness in dairy cattle, imposing welfare and profitability concerns. Low to moderate heritability estimates of SU and WLD susceptibility indicate that genetic selection could reduce their prevalence. To identify the susceptibility loci for SU, WLD, SU and/or WLD, and any type of noninfectious claw lesion, genome-wide association studies (GWAS) were performed using generalized linear mixed model (GLMM) regression, chunk-based association testing (CBAT), and a random forest (RF) approach. Cows from five commercial dairies in California were classified as controls having no lameness records and ≥6 years old (*n* = 102) or cases having SU (*n* = 152), WLD (*n* = 117), SU and/or WLD (SU + WLD, *n* = 198), or any type of noninfectious claw lesion (*n* = 217). The top single nucleotide polymorphisms (SNPs) were defined as those passing the Bonferroni-corrected suggestive and significance thresholds in the GLMM analysis or those that a validated RF model considered important. Effects of the top SNPs were quantified using Bayesian estimation. Linkage disequilibrium (LD) blocks defined by the top SNPs were explored for candidate genes and previously identified, functionally relevant quantitative trait loci. The GLMM and CBAT approaches revealed the same regions of association on BTA8 for SU and BTA13 common to WLD, SU + WLD, and NICL. These SNPs had effects significantly different from zero, and the LD blocks they defined explained a significant amount of phenotypic variance for each dataset (6.1–8.1%, *p* < 0.05), indicating the small but notable contribution of these regions to susceptibility. These regions contained candidate genes involved in wound healing, skin lesions, bone growth and mineralization, adipose tissue, and keratinization. The LD block defined by the most significant SNP on BTA8 for SU included a SNP previously associated with SU. The RF models were overfitted, indicating that the SNP effects were very small, thereby preventing meaningful interpretation of SNPs and any downstream analyses. These findings suggested that variants associated with various physiological systems may contribute to susceptibility for NICL, demonstrating the complexity of genetic predisposition.

## Introduction

Lameness, or abnormal gait and/or posture, is a pathognomonic sign that the affected cow is in pain and frequently reflects claw damage. Many claw conditions can cause lameness, including injury, infectious claw lesions, and noninfectious claw lesions. The two most common noninfectious claw lesions causing lameness in dairy cattle are sole ulcers (SUs), also known as pododermatitis circumscripta, and white line disease (WLD) (Green et al., 2002; Shearer and van Amstel, 2017). These lesions are not only a welfare issue but are also associated with reduced milk production and decreased fertility (Green et al., 2002, 2010; Hernandez et al., 2005; Charfeddine and Pérez-Cabal, 2017). Consequently, SU and WLD represent a considerable financial burden, with the average costs associated with prevention, treatment, and losses from reduced productivity ranging from $181 (Dolecheck et al., 2019) to $258 (Cha et al., 2010) per case of SU and $155 for WLD (Dolecheck et al., 2019) (adjusted to 2020 US dollars). Production losses from extended calving interval, increased culling, and decreased milk production increase greenhouse gas emissions by 33 (3.6%) and 39 (4.3%) kg CO_2_ equivalents per ton of fat- and protein-corrected milk per case of SU and WLD, respectively (Mostert et al., 2018). Reducing the prevalence of SU and WLD would alleviate these welfare, economic, and environmental concerns and thereby improve the sustainability of dairy production.

Both genetic and non-genetic factors contribute to susceptibility to SU and WLD, and prevention can be achieved through genetic means and herd management. Current prevention methods focus on management control primarily through regular claw trimming (Shearer and van Amstel, 2001) and providing rubber flooring in stalls and alleys (Vanegas et al., 2006; Fjeldaas et al., 2011; Eicher et al., 2013). Although dairies have implemented these prevention methods, SU and WLD remain prevalent worldwide, with estimates ranging from 4.1 to 27.8% for SU and from 2.0 to 11% for WLD in Holstein cattle depending on parity and the housing style (Cramer et al., 2008; Bicalho et al., 2009; van der Linde et al., 2010; Oberbauer et al., 2013). Heritability estimates of susceptibility range from 0.01 to 0.3 for SU and from 0.017 to 0.26 for WLD (Van der Waaij et al., 2005; van der Linde et al., 2010; Häggman and Juga, 2013; Oberbauer et al., 2013; van der Spek et al., 2013, 2015a; Malchiodi et al., 2015a), implying that these non-genetic means to reduce prevalence could be bolstered by genetic selection against susceptibility to these claw lesions. Although many genome-wide association studies (GWAS) have been performed to identify the susceptibility loci, loci previously associated with SU and WLD are discordant (Malchiodi et al., 2015b; van der Spek et al., 2015b; Sánchez-Molano et al., 2019), and susceptibility to these claw lesions is believed to be a complex trait governed by loci of small effect (van der Spek et al., 2015b). Some have postulated that selection against susceptibility to SU, WLD, and other noninfectious claw lesions could be achieved through indirect selection on body conformation traits or feet and leg traits (Van der Waaij et al., 2005; Häggman et al., 2013). However, the genetic correlation between the conformation traits and susceptibility to noninfectious claw lesions appears to be low (Häggman and Juga, 2013; Malchiodi et al., 2015b; Ring et al., 2018), further accentuating the need to identify loci associated directly with susceptibility to noninfectious claw lesions. Thus, the objective of this study was to identify the genomic regions associated with susceptibility to SU, WLD, SU and/or WLD, and noninfectious claw lesions using well-characterized herds under similar management practices: we hypothesized that we would identify small-effect loci associated with predisposition to noninfectious claw lesions in addition to those already identified.

## Materials and Methods

All procedures were conducted in accordance with the ethical standards set by the University of California, Davis, and approved by the Institutional Animal Care and Use Committee (protocol no. 22099).

### Phenotypic Data

Dairies were selected to minimize environmental variations by including dairies in Central and Northern California using freestall housing, a flush system for waste removal, and diets balanced to meet the nutrition requirements from the National Research Council (National Research Council (NRC), 2001). Case/control phenotypes were defined using hoof trimming records. The hoof trimming records were generated by three hoof trimmers: one serviced dairies A, B, and C; one serviced dairy D; and the last trimmer serviced dairy E. Hoof trimmer qualifications were described in a previous paper (Lai et al., 2020), and the three trimmers employed common criteria in defining the lesions. Hoof trimming regimens varied among dairies: cows were trimmed at the beginning of and at mid-lactation, at dry off, and when lame (dairy A); at dry off and when lame (dairies B and C); only when lame (dairy D); and at mid-lactation, at dry off, and when lame (dairy E). The following claw lesions were documented in the hoof trimming records: SU, hemorrhage, sole fracture, sole abscess, wall abscess, white line abscess (WLD), heel abscess, laminitis, foot wart, and foot rot. Cows were phenotyped as cases or controls based on whether they had or lacked records of claw lesions, respectively. Four case/control datasets were generated based on the type(s) of claw lesions the cases had. For datasets 1 (SU) and 2 (WLD), cases were defined as cows with at least one record of SU or WLD, respectively. For dataset 3 (SU + WLD), cases included cows with either one or both of the claw lesions. Cases for dataset 4 (noninfectious claw lesions, NICL) included cows with at least one of the following noninfectious claw lesions: SU, hemorrhage, sole fracture, sole abscess, wall abscess, WLD, heel abscess, and/or laminitis. Cows with no claw lesions and that were at least 6.0 years old were considered sound controls. The age restriction was imposed to avoid misphenotyping younger cows that had insufficient time to develop claw lesions. The same sound controls were used to compare against the cases in each of the four datasets.

### Genotypes

Whole blood was collected from cows phenotyped as cases and controls. DNA was extracted from whole blood samples using the QIAGEN QIAamp DNA Blood Mini Kit (QIAGEN Inc., Valencia, CA) and quantified using the NanoDrop (ND-2000 v3.2.1) spectrophotometer (Thermo Scientific, Wilmington, DE, United States). DNA samples were genotyped on the BovineHD BeadChip [777K single nucleotide polymorphisms (SNPs), Illumina Inc., San Diego, CA, United States] by GeneSeek (Lincoln, NE, United States), and Illumina’s GenCall algorithm was used to call genotypes. A portion of the controls used in this study were the same controls used in our previous study (Lai et al., 2020), for which raw and processed genotype data are publicly available at the NCBI Gene Expression Omnibus database (GEO series record GSE159157). Additional cows genotyped in this study are available in the GEO database (GEO series record GSE165945).

Genotypes were updated to the ARS-UCD1.2 assembly positions (Rosen et al., 2020) and quality filtered using PLINK 1.9 (Chang et al., 2015; Purcell and Chang, 2015) to remove from further analyses SNPs and cows with genotyping rates <95%, SNPs with significant deviation from Hardy–Weinberg equilibrium (*p* < 1E−6) to exclude systematic genotyping errors, and SNPs with minor allele frequencies (MAFs) < 5% to exclude rare variants. To visualize genetic similarity among the remaining cows, multidimensional scaling (MDS) analysis was performed, and the first two dimensions were plotted. Because the downstream programs for GWAS analysis [the generalized linear mixed model (GLMM) and random forest (RF)] required genotypes at each SNP, missing genotypes remaining after quality filtering were imputed using BEAGLE 5.1 (Browning et al., 2018) using the default parameters and an effective population size of 58 previously estimated for North American Holstein cattle (Makanjuola et al., 2020).

### Generalized Linear Mixed Model GWAS

Because disease phenotype was binary (cases and controls), the model used for association testing needed to reflect this binary outcome. Accordingly, logistic regression was used to model the binary outcome for the power analysis and for association testing. Power analysis was conducted using the genpwr R package (Moore et al., 2019), assuming an additive genetic effect and a sample size and case rate similar to the sample population (sample size = 275, case rate = 0.6). Given these parameters, the smallest effect SNP that the GWAS was expected to detect would have an odds ratio of at least 1.7 and a MAF of at least 0.34. For association testing, a genetic relatedness matrix (GRM) and farms were included as covariates in the model to account for population stratification and relatedness as well as the effect of farm, respectively. The probability of disease was defined as *p*_*ijk*_ for the *k*-th cow on the *i*-th farm identified in the *j*-th SNP genotype class and the logit of this probability, as θ_*ijk*_ = log[*p*_*ijk*_/(1−*p*_*ijk*_)]. The logit of the probability of disease was modeled as a function of the recorded explanatory variables (e.g., farm and SNP genotype) along with a presumed quantitative genetic contribution for each SNP:

$$\theta_{i\,j\,k}=\mu +F_{i}+S_{j}+a_{k}$$

where μ is an unknown constant common to all cows, *F*_*i*_ the contribution of *i*-th farm to the risk of disease, and *S*_*j*_ is the contribution of the *j*-th SNP genotype to the risk of disease. The additive genetic effect *a*_*k*_ is assumed to be drawn from the multivariate normal density *N*(0, $A\,\sigma_{a}^{2}$), with *A* as the standardized GRM among the animals in the dataset calculated in GEMMA (Zhou and Stephens, 2012) and $\sigma_{a}^{2}$ is the unknown additive genetic variance of the disease risk. Model fitting and association testing via the score test (i.e., the Legrange multiplier test) were implemented with the generalized linear mixed model association test (GMMAT) R package (Chen et al., 2016).

The effective number of independent markers (*M*_*e*_) was calculated as the number of SNPs remaining after linkage disequilibrium (LD) pruning using the Genetic Type I error calculator and used as the denominator for Bonferroni correction of the association *p* values (Li et al., 2012). Significant SNPs were defined as those with *p* ≤ 0.05/*M*_*e*_ and suggestive SNPs were defined as those with *p* ≤ 1/*M*_*e*_ (Lander and Kruglyak, 1995). Genomic inflation factors were calculated as the ratio of the median of the observed and expected *p* values. Quantile–quantile plots (qqplots) and Manhattan plots were plotted using the R package *qqman* (R Development Core Team, 2010; Turner, 2014).

### Chunk-Based Association Testing

Chunk-based association testing (CBAT), also called set-based association testing, was performed to decrease multiple testing and, in turn, improve the power of detecting associated regions in the small sample size. In contrast to gene-based association testing, which jointly tests variants within genes for association with the phenotype (e.g., Xia et al., 2017), CBAT analyzes consecutive windows of variants (i.e., chunks) across each chromosome without prior filtering. Accordingly, CBAT includes variants in non-coding regions containing regulatory elements that could contribute to phenotypic variation in complex traits (Koufariotis et al., 2014, 2018). Quality-filtered SNPs were split into 100-kb chunks overlapping by 50 kb. Each chunk was LD-pruned to remove SNPs that were in strong LD (*R*^2^ > 0.98) and then tested for association with the phenotype by determining whether the phenotypic variance explained (PVE) by the chunk was significantly greater than zero. Specifically, association testing for each chunk was performed by calculating a GRM using the SNPs in the chunk and regressing the phenotype on the GRM. In addition to the chunk-based GRM, a thinned GRM (from genome-wide SNPs) and farms were included as covariates in the model to adjust for population stratification and differences among farms. The thinned GRM was calculated using genome-wide LD-pruned SNPs: SNPs within a window of 1 Mb and a *R*^2^ > 0.5 were pruned out such that only SNPs in linkage equilibrium were used in the GRM calculation. For each chunk of SNPs, the following linear model was used to define the disease phenotype *y* for the *k*-th cow as a function of phenotypic contribution from the *j*-th chunk that comprised *m* SNPs and the *i*-th farm:

$$y_{i\,j\,k}=\mu +F_{i}+C_{j}+a_{k}+\varepsilon_{i\,j\,k}$$

where μ, *F*_*i*_, and *a*_*k*_ are the same components outlined in the previous equation contributing to phenotype (coded as 0 for controls and 1 for cases), $C_{j}=\sum_{l=1}^{m}S_{l}$ is the contribution of the chunk to the phenotype in which *S*_*l*_ is the contribution of the *l*-th SNP in the chunk, and ε_*ijk*_ is the residual term. Estimates of PVE for each chunk were transformed to the underlying liability scale to adjust for ascertainment of cases using prevalence estimates from the literature: 4.08% for SU, 7.89% for WLD, 0.10 for SU + WLD, and 0.10 for NICL (DeFrain et al., 2013; Oberbauer et al., 2013). Calculating the thinned GRM, estimating PVE by each chunk, association testing with the likelihood ratio test, and *p* value estimation via 10 permutations for each chunk (Listgarten et al., 2013) were performed using the linkage disequilibrium-adjusted kinships (LDAK) program (Speed et al., 2012). For each dataset, the significance thresholds were adjusted using Bonferroni correction: chunks with *p* ≤ 0.05/(number of chunks) were defined as significant and chunks with *p* ≤ 1/(number of chunks) were defined as suggestive (Lander and Kruglyak, 1995). Manhattan plots and qqplots were plotted using the R package *qqman* (R Development Core Team, 2010; Turner, 2014).

### Random Forest GWAS

A RF fits a model that includes all SNPs and does not require an assumption about the mode of inheritance (e.g., additive, dominant, and recessive), making RFs an appealing approach for complex traits such as susceptibility to claw lesions, in which the trait is highly polygenic and epistasis is present (Goldstein et al., 2010). Furthermore, RFs are insensitive to uneven sampling of cases and controls across different dairies, as RFs first build decision trees, then quantify the importance values afterward with data available in the trees.

Linkage disequilibrium pruning and RF analyses were performed as previously detailed (Lai et al., 2020) for each of the four datasets. Briefly, LD-pruned genotypes and farms were used as predictors for the RF analyses performed using the *caret* R package (Kuhn, 2008; R Development Core Team, 2010). For each dataset, the population was randomly divided into a training (two-thirds of the cows) and a test (one-third of the cows) population. Using the training population, the number of predictors considered at each node of each decision tree, *mtry*, was tuned using five values, 0.1*p*, 0.2*p*, 0.5*p*, 0.8*p*, and *p*, where *p* is the total number of predictors (Goldstein et al., 2010; Brieuc et al., 2018). The *mtry* resulting in the most accurate RF model was used for downstream analyses. The most important predictor was assigned a value of 100, and any other predictor’s importance values was scaled accordingly (e.g., a predictor with an importance value of 50 is 50% as important as the most important predictor). Model validation was performed by using the predictors and their importance values to predict the case/control phenotype in the test population. To determine which SNPs were important and worthy of further investigation, a scree plot was plotted and the second-order point of inflection was identified using the inflection R package (Christopoulos, 2016, 2017) (i.e., the “elbow method”). Predictors with importance values equal to or greater than the second-order point of inflection were defined as important SNPs and explored in downstream analyses if and only if the RF model was significantly more accurate at predicting phenotype in the test population than the non-information rate (i.e., the frequency of the more common phenotype).

### Defining Associated Regions

For each of the four datasets, the top SNPs were defined as significant and suggestive SNPs from the GLMM regression or important SNPs from a significantly predictive RF model. Boundaries of the genomic regions of association were defined using SNPs in LD with top SNPs. Similar to the methodology of Richardson et al. (2016) and Twomey et al. (2019), the positions of SNPs within 5 Mb and with *R*^2^ ≥ 0.5 of each top SNP were determined using non-pruned imputed genotypes, and the furthest SNP upstream and downstream in LD with the significant or suggestive SNP defined the LD block boundaries. Overlapping LD blocks were combined. Using the same procedure outlined for CBAT, the PVE by the LD blocks defined from the GLMM and RF analyses was estimated and compared against the PVE by chunks of SNPs of the same size that overlapped by 50 kb from all chromosomes.

### Bayesian Estimation of SNP Effects and Assessing Model Fit

A Bayesian approach was used to test the association of the top SNPs identified in the GLMM and the RF with the case/control phenotype for the four datasets. Bayesian methodology was selected because it allows multiple SNPs to be fitted jointly, recognizes that some SNPs are correlated and most likely have small effects on susceptibility (van der Spek et al., 2015b), and can account for the uneven sampling of cases and controls from dairies. Additionally, the effect size estimates obtained from Bayesian estimation are directly interpretable, and Bayesian model evaluation is extremely thorough. Because highly correlated predictors complicate Bayesian regression, the significant and suggestive SNPs detected in the GLMM GWASs were LD-pruned (*R*^2^ > 0.9) using PLINK 1.9 (Chang et al., 2015; Purcell and Chang, 2015) prior to estimating effects to keep the most significant SNP in each LD block for inclusion into the Bayesian model. Estimation of SNP effects was performed using a Bayesian logistic regression model as described in Lai et al. (2020). The important SNPs from the RF did not need to be LD-pruned, as SNPs were LD-pruned prior to RF analyses. Briefly, each set of top SNPs (i.e., LD-pruned suggestive/significant SNPs from the GLMM analyses and important SNPs from RF analyses) was used as predictors along with farm as a covariable in a Bayesian logistic regression model, and the model was fitted via sampling the posterior using the Hamiltonian Monte Carlo algorithm in the R package *rstanarm* (Gelman et al., 2020; Goodrich et al., 2020). The same population was used in the GLMM and RF GWAS as for the SNP effect estimation, which could lead to the inclusion of false-positive associations in the Bayesian model. Thus, to discern whether the included SNPs were false positives, the fit of the Bayesian model using the estimated parameters was evaluated using leave-one-out (LOO) cross-validation and posterior predictive checking (PPC) using the *loo* and *bayesplot* R packages (Vehtari et al., 2017, 2020; Gabry et al., 2019). Bayesian estimation of the SNP effects generated a distribution of where the true value of the SNP effect was, and this range was quantified in the 95% uncertainty intervals (UI), as opposed to a point estimate in frequentist methods. SNPs with 95% UIs that did not overlap zero were considered significantly associated with susceptibility to the respective claw lesion(s).

### Functional Annotation of Associated Regions

Genes and previously defined quantitative trait loci (QTL) falling within or overlapping with the associated LD blocks and chunks were obtained using FAANGMine using the genomic regions search function (Functional Annotation of Animal Genomes (FAANG), 2019) and the CattleQTLdb (Hu et al., 2019). RefSeq genes were extracted from the resulting gene list and used in the pathway and gene ontology enrichment analysis in FAANGMine. Genes were searched in the Mouse Genome Informatics batch query database to find the associated mammalian phenotypes (Smith and Eppig, 2009). Genes were also queried in the Cattle Gene Atlas (Fang et al., 2020) to determine in which tissues they were expressed.

## Results

### Descriptive Data

The percentage and count of cows with records of each claw lesion from each dairy are presented in Table 1. Of the cows that had hoof trimming records from the five dairies, 5.6 and 12.0% had records of SU and WLD, respectively, similar to previous prevalence estimates (Cramer et al., 2008; Bicalho et al., 2009; van der Linde et al., 2010; Oberbauer et al., 2013). For cows that were genotyped, cases were sampled from all five dairies, whereas controls were sampled from dairies A and D, which had cows that met our strict soundness and age criteria for controls. The dataset included 156 SU cases, 119 WLD cases, 203 SU + WLD cases (72 cows had both SU and WLD), 222 NICL cases, and 104 sound controls, for a total of 287 cows (Table 2). The average age of the controls sampled was 8.7 years old (SD = 1.4), and when compared to the average age of onset of 4.2 (SD = 1.7) for SU and 4.5 (SD = 2.6) years for WLD, it indicated that our age cutoff of 6.0 years old was sufficient to avoid misphenotyping control cows.

**TABLE 1:** Percent (and number) of cows with records of foot lesions across the five dairies.

Farm	Percentage of cows with lesion (*n*)										
	Sole ulcer	White line disease	Foot wart	Wall abscess	Sole abscess	Sole fracture	Foot rot	Bruise	Heel abscess	Severe laminitis	Total no. of cows with records
A	6.9 (467)	15.6 (1050)	5.6 (376)	2.2 (146)	4.7 (319)	1.9 (125)	0.9 (62)	NR	NR	NR	6,734
B	1.2 (44)	2.6 (91)	8.5 (301)	0.2 (6)	0.6 (23)	1 (37)	0 (1)	NR	NR	NR	3,549
C	1.3 (35)	2.1 (57)	8.8 (236)	0.5 (13)	0.3 (9)	0.4 (10)	0 (1)	NR	NR	NR	2,676
D	5.5 (254)	12.8 (596)	5.8 (268)	NR	NR	NR	0.9 (42)	1.6 (73)	NR	NR	4,658
E	11.1 (380)	21.4 (733)	17.9 (614)	NR	NR	NR	1.1 (39)	NR	16.3 (559)	8.3 (284)	3,427
Total^a^	5.6 (1180)	12.0 (2527)	8.5 (1795)	1.3 (165)	2.7 (351)	1.3 (172)	0.7 (145)	1.6 (73)	16.3 (559)	8.3 (284)	21,044

*NR, not recorded. ^*a*^Totals were calculated across dairies that had records of the lesion; dairies that did not record the lesion were excluded from the calculation of totals.*

**TABLE 2:** Distribution of cases for sole ulcers (SU), white line disease (WLD), SU + WLD, noninfectious claw lesions (NICL), and sound controls after quality filtering across the five dairies.

Farm	Controls	Cases			
		SU	WLD	SU + WLD	NICL
A	81	44	48	75	87
B	0	8	13	17	23
C	0	4	7	9	10
D	21	71	33	72	72
E	0	25	16	25	25
Total	102	152	117	198	217

After quality filtering, ∼556,000 SNPs for 152 SU cases, 117 WLD cases, 198 SU + WLD cases (71 cases had both SU and WLD), 217 NICL cases, and 102 sound controls remained for MDS, GLMM, Genetic Type I error calculation, CBAT, and RF analyses. The MDS plot showed some population stratification, with a prominent center cluster and two other sparse clusters, although clustering was not by farm or case/control phenotype (Supplementary Figure 1). Pairwise relationship coefficients calculated for the GRM ranged from −0.094 to 0.50, with negative values indicating that the two cows were less related to each other than other random pairs of individuals. The distribution of the pairwise relationship coefficients did not differ greatly between pairs of cows from the same farm and pairs from different farms (Supplementary Figure 2). The Genetic Type I error calculator determined that the effective number of markers on autosomal chromosomes for Bonferroni correction was ∼156,000 SNPs for the four datasets, yielding a significance threshold of *p* = 3.2 × 10^–7^ [6.5 on −log_10_(*p*) scale] and a suggestive threshold of *p* = 6.4 × 10^–6^ [5.2 on −log10(*p*) scale]. The total number of 100-kb chunks used in CBAT was ∼51,730 for the four datasets, yielding a significance threshold of *p* = 9.7 × 10^–7^ [6.0 on −log_10_(*p*) scale] and a suggestive threshold of *p* = 1.9 × 10^–5^ [4.7 on −log_10_(*p*) scale]. Linkage disequilibrium pruning at *R*^2^ > 0.90 left 215,343–218,185 SNPs for RF analysis, depending on the dataset.

### Generalized Linear Mixed Model GWAS and CBAT

The GLMM analyses detected a region of association on BTA8 for SU and BTA13 for WLD, SU + WLD, and NICL while sufficiently accounting for population stratification and relatedness, as indicated by the qqplots and the genomic inflation factors of 1.01, 1.02, 1.01, and 0.99 for SU, WLD, SU + WLD, and NICL, respectively (Supplementary Figure 3). The CBAT using 100-kb overlapping chunks across the genome also properly accounted for population stratification and relatedness (qqplots in Supplementary Figure 5) and identified the same regions as the single-marker GLMM GWAS for each of the four datasets, providing further support for these regions (Supplementary Table 1; Manhattan plots in Supplementary Figure 6). The SU CBAT also identified two suggestive chunks on BTA17 (Supplementary Table 1 and Supplementary Figure 6A). For the NICL CBAT, the reduction in the number of tests performed allowed the chunk at BTA13:46,450,001–46,550,001 to reach genome-wide significance (*p* = 6.9 × 10^–7^; Supplementary Table 1 and Supplementary Figure 6D). This significant chunk contained the most significant SNP from the single-marker GLMM GWAS and three suggestive SNPs downstream.

**FIGURE 1:**
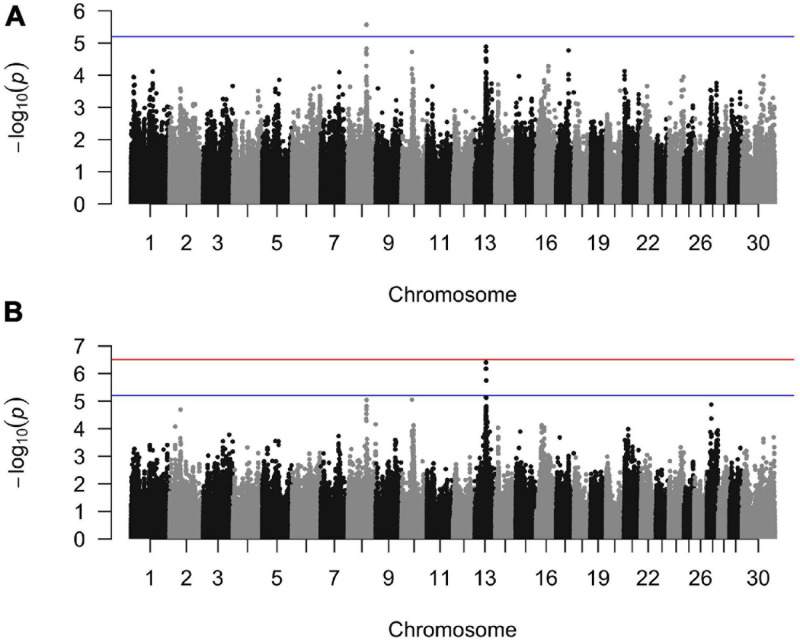
Manhattan plots from the generalized linear mixed model regression association analyses for **(A)** sole ulcers and **(B)** noninfectious claw lesion susceptibility. The *blue line* indicates the threshold of genome-wide suggestive significance and the *red line* indicates the threshold of genome-wide significance.

**FIGURE 2:**
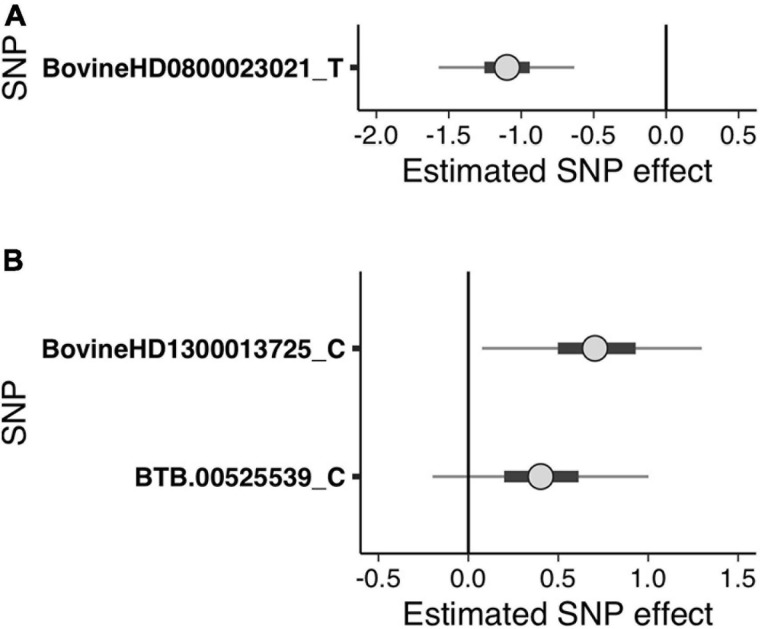
Bayesian uncertainty interval (UI) plots depicting the estimated single nucleotide polymorphism (SNP) effects of the suggestive SNPs detected in the generalized linear mixed model regression analysis for **(A)** sole ulcers and **(B)** noninfectious claw lesion susceptibility. *Dots* indicate the median of the SNP effect, *thick black bars* indicate the 50% UI, and *thin lines* indicate the 95% UI of the effect size distribution. The *letters following SNP names* indicate the minor allele for which the effect was calculated. Positive values indicate that the minor allele of the SNP increases susceptibility, and negative values indicate that the minor allele of the SNP decreases susceptibility.

The GLMM association testing for SU susceptibility identified 12 suggestive SNPs on BTA8 falling in or directly upstream of the gene *DCAF12* (also known as DDB1 and CUL4-associated factor 12) (Tables 3, 4). The 12 suggestive SNPs collectively defined a 3.2-Mb LD block at BTA8:74,345,807–77,546,693 (Table 3 and Figure 1A) encompassing or overlapping with 60 genes: 52 protein-coding genes, four long non-coding RNA (lncRNA) genes, a transfer RNA (tRNA) gene, a microRNA (miRNA) gene, a small nuclear RNA (snRNA) gene, and a small nucleolar RNA (snoRNA) gene. Because the 12 suggestive SNPs from the SU GLMM were in strong LD (*R*^2^ > 0.9), the most significant SNP, BovineHD0800023021, was selected to represent this LD block in the Bayesian logistic regression model. The minor allele at BovineHD0800023021 (T) had an effect that was significantly less than zero at 95% UI (Table 3 and Figure 2A), indicating that it was associated with reduced susceptibility to SU. The LOO analysis yielded acceptable Pareto *k* values (*k* < 0.5) for all cows, which indicated that the model was able to predict the phenotype of each cow with similar accuracy using the genotypes at BovineHD0800023021 from all other cows. Goodness-of-fit assessment via PPC also showed that the distribution of the phenotypes simulated using the estimated SNP effect closely aligned with that of the observed data (Supplementary Figure 7A), further validating the fit of the model. In addition to identifying suggestive chunks in the same regions on BTA8, CBAT for SU detected two significant chunks on BTA17 (Supplementary Table 1 and Supplementary Figure 6A) that both fell within *TMEM12* (transmembrane protein 132B).

**TABLE 3:** SNPs that were suggestive in the generalized linear mixed model association analysis and the linkage disequilibrium (LD) blocks they defined for sole ulcers (SU), white line disease (WLD), sole ulcers and/or white line disease (SU + WLD), and noninfectious claw lesions (NICL).

Dataset	BTA	SNP	SNP position (bp)	Minor/major allele	Minor allele count		Minor allele frequency		Score^a^ (variance)	*p*	SNP significance in Bayesian estimation^b^	LD block start (bp)	LD block end (bp)	LD block length (kb)
					Cases	Controls	Cases	Controls						
SU	8	BovineHD0800023014	75,489,164	T/C	75	94	0.247	0.461	−20 (18.1)	2.71E−06	–	74,345,807	77,546,693	3,200.9
	8	BovineHD0800023015	75,490,011	T/G	75	94	0.247	0.461	−20 (18.1)	2.71E−06	–	74,345,807	77,546,693	3,200.9
	8	ARS-BFGL-NGS-112795	75,490,692	A/G	75	94	0.247	0.461	−20 (18.1)	2.71E−06	–	74,345,807	77,546,693	3,200.9
	8	BovineHD0800023016	75,491,531	C/T	75	94	0.247	0.461	−20 (18.1)	2.71E−06	–	74,345,807	77,546,693	3,200.9
	8	BovineHD0800023017	75,492,307	G/A	75	94	0.247	0.461	−20 (18.1)	2.71E−06	–	74,345,807	77,546,693	3,200.9
	8	BovineHD0800023018	75,493,464	T/C	75	94	0.247	0.461	−20 (18.1)	2.71E−06	–	74,345,807	77,546,693	3,200.9
	8	BovineHD0800023019	75,494,163	C/T	75	94	0.247	0.461	−20 (18.1)	2.71E−06	–	74,345,807	77,546,693	3,200.9
	8	BovineHD0800023021	75,496,244	T/C	77	97	0.253	0.476	−20.4 (18.8)	2.66E−06	*	74,345,807	77,546,693	3,200.9
	8	BovineHD0800023022	75,496,918	A/G	75	94	0.247	0.461	−20 (18.1)	2.71E−−06	–	74,345,807	77,546,693	3,200.9
	8	BovineHD0800023023	75,497,471	C/T	75	94	0.247	0.461	−20 (18.1)	2.71E−06	–	74,345,807	77,546,693	3,200.9
	8	BovineHD0800023024	75,498,118	A/G	75	94	0.247	0.461	−20 (18.1)	2.71E−06	–	74,345,807	77,546,693	3,200.9
	8	BovineHD0800023025	75,501,482	T/C	75	94	0.247	0.461	−20 (18.1)	2.71E−06	–	74,345,807	77,546,693	3,200.9
WLD	13	BovineHD1300013725	46,491,619	C/T	106	48	0.453	0.235	19.9 (19.4)	6.13E−06	*	46,307,416	47,584,595	1,277.2
SU + WLD	13	BovineHD1300013725	46,491,619	C/T	183	48	0.462	0.235	25 (25.5)	7.03E−07	*	45,283,136	47,676,681	2,393.5
	13	BovineHD1300013733	46,526,509	C/T	188	52	0.475	0.255	24.8 (25.6)	9.86E−07	–	45,283,136	47,676,681	2,393.5
	13	BovineHD1300013739	46,540,186	G/T	188	52	0.475	0.255	24.8 (25.6)	9.86E−07	–	45,283,136	47,676,681	2,393.5
	13	BovineHD1300013740	46,541,925	C/T	188	52	0.475	0.255	24.8 (25.6)	9.86E−07	–	45,283,136	47,676,681	2,393.5
	13	BovineHD1300013750	46,561,964	C/T	188	52	0.475	0.255	24.8 (25.6)	9.86E−07	–	45,283,136	47,676,681	2,393.5
	13	BovineHD1300013759	46,582,769	G/A	188	52	0.475	0.255	24.8 (25.6)	9.86E−07	–	45,283,136	47,676,681	2,393.5
	13	BovineHD1300013765	46,596,264	A/G	188	52	0.475	0.255	24.8 (25.6)	9.86E−07	–	45,283,136	47,676,681	2,393.5
	13	BovineHD1300013774	46,637,235	A/G	188	52	0.475	0.255	24.8 (25.6)	9.86E−07	–	45,283,136	47,676,681	2,393.5
	13	BTB-00525539	47,420,271	C/A	195	59	0.492	0.289	24.6 (27.8)	3.03E−06	ns	45,283,136	47,676,681	2,393.5
NICL	13	BovineHD1300013725	46,491,619	C/T	199	48	0.459	0.235	26.4 (27.2)	3.96E−07	*	45,283,136	47,676,681	2,393.5
	13	BovineHD1300013733	46,526,509	C/T	204	52	0.470	0.255	26 (27.3)	6.68E−07	–	45,283,136	47,676,681	2,393.5
	13	BovineHD1300013739	46,540,186	G/T	204	52	0.470	0.255	26 (27.3)	6.68E−07	–	45,283,136	47,676,681	2,393.5
	13	BovineHD1300013740	46,541,925	C/T	204	52	0.470	0.255	26 (27.3)	6.68E−07	–	45,283,136	47,676,681	2,393.5
	13	BovineHD1300013750	46,561,964	C/T	204	52	0.470	0.255	26 (27.3)	6.68E−07	–	45,283,136	47,676,681	2,393.5
	13	BovineHD1300013759	46,582,769	G/A	204	52	0.470	0.255	26 (27.3)	6.68E−07	–	45,283,136	47,676,681	2,393.5
	13	BovineHD1300013765	46,596,264	A/G	204	52	0.470	0.255	26 (27.3)	6.68E−07	–	45,283,136	47,676,681	2,393.5
	13	BovineHD1300013774	46,637,235	A/G	204	52	0.470	0.255	26 (27.3)	6.68E−07	–	45,283,136	47,676,681	2,393.5
	13	BTB-00525539	47,420,271	C/A	213	59	0.491	0.289	25.8 (29.3)	1.79E−06	ns	45,283,136	47,676,681	2,393.5

^*a*^Scores are for the minor allele generated from the generalized linear mixed model analysis. Negative scores indicate that the minor allele is associated with reduced susceptibility and positive scores indicate that the minor allele is associated with increased susceptibility. ^*b*^SNPs used in the Bayesian model were either significantly different from zero at 95% uncertainty interval (*) or not significant (ns). Other SNPs were in LD with SNPs that were used in the model and were excluded from the model (–).

**TABLE 4:** Proportion of phenotypic variance explained (PVE) by each linkage disequilibrium (LD) block defined from the generalized linear mixed model association analysis compared to the mean PVE of all chunks of genomic regions with the same length for sole ulcers (SU), white line disease (WLD), sole ulcers and/or white line disease (SU + WLD), and noninfectious claw lesions (NICL).

	LD block						Genome-wide mean of chunks with same length as LD block	
Dataset	BTA	Start (bp)	End (bp)	Length (kb)	PVE (SD)	PVE *p*	PVE (SE)	PVE *p* (SE)
SU	8	74,345,807	77,546,693	3,200.90	0.081 (0.054)	3.93E−04	0.00809 (0.0004)	0.478 (0.006)
WLD	13	46,307,416	47,584,595	1,277.20	0.061 (0.047)	2.93E−05	0.00794 (0.0002)	0.485 (0.004)
SU + WLD	13	45,283,136	47,676,681	2,393.50	0.071 (0.050)	1.05E−06	0.00873 (0.0004)	0.482 (0.006)
NICL	13	45,283,136	47,676,681	2,393.50	0.074 (0.051)	5.79E−09	0.00828 (0.0003)	0.484 (0.005)

For WLD, the GLMM association testing found a single suggestive intergenic SNP at BTA13:46,491,619 (BovineHD1300013725; Supplementary Figure 4A), which was also the most significant SNP identified by the GLMM analyses for SU + WLD and NICL (Table 3 and Supplementary Figure 4B and Figure 1B). In addition to detecting BovineHD1300013725, the GLMM analyses for the SU + WLD and NICL datasets detected eight other suggestive SNPs in the same LD block as BovineHD1300013725 (Table 3 and Supplementary Figure 4). These nine suggestive SNPs detected in the SU + WLD GWAS were slightly more significant in the NICL GWAS and defined a 2.4-Mb LD block at BTA13:45,283,136–47,676,681 containing 27 genes: 16 protein-coding genes, six lncRNA genes, two snRNA genes, two snoRNA genes, and one miRNA gene. For all four GLMM GWAS, the limited number of genes in the LD blocks defined from suggestive SNPs precluded pathway and gene ontology analyses.

Given that the GLMM GWAS for SU + WLD and NICL identified nine suggestive SNPs in the same LD block (*R*^2^ > 0.5) on BTA13 (Figure 1B and Supplementary Figure 4) and the top SNP is the same as that in the WLD GWAS, only the NICL Bayesian SNP effect estimation results are presented. Eight of these suggestive SNPs were in strong LD (*R*^2^ > 0.9), whereas the remaining suggestive SNP (BTB-00525539) was in weaker LD with the others (*R*^2^ = 0.7). Consequently, the most significant SNP in the LD block of eight SNPs (BovineHD1300013725) and BTB-00525539 were included in the Bayesian logistic regression model. The minor allele at BovineHD1300013725 representing the eight SNPs in strong LD had an effect that was significantly greater than zero at 95% UI (Figure 2B), indicating that the minor allele (C) was associated with increased susceptibility to NICL (Table 3). In contrast, the effect of the minor allele at BTB-00525539 was not significantly different from zero (Figure 2B). Although the score variances of the suggestive SNPs were large (Table 3), possibly due to the sample cohort, Bayesian estimation was less affected than GLMM regression by these limitations and indicated that the SNP effects were significant for SU and NICL (Figure 2). For the LOO analysis of the model, the acceptable Pareto *k* values (*k* < 0.5) from all cows demonstrated that the model including BovineHD1300013725 and BTB-00525539 was able to predict the NICL phenotype of each cow based on the genotypes at these two SNPs from the other cows with similar accuracy. The PPC-simulated data based on the estimated SNP effects of these two SNPs were similar to the observed data, indicating good model fit (Supplementary Figure 7B).

To draw attention to the impactful SNPs shown in Table 3 and the LD blocks they defined in Table 4, the minor allele frequencies at the most significant SNP for SU (BovineHD0800023021) in cases and controls were 0.253 and 0.476, respectively. The GLMM output score was negative and Bayesian estimation indicated a significant negative effect on susceptibility; that is, the minor allele was associated with reduced susceptibility. In contrast, the MAF at the most significant SNP for NICL (BovineHD1300013725) was higher in cases (0.459) than in controls (0.235), indicating that the minor allele was associated with higher susceptibility. Likewise, the GLMM score was positive, and Bayesian estimation of the effect size resulted in a significant positive effect. Similar minor allele frequencies, scores, and significantly positive effect size estimates were observed at BovineHD1300013725 for WLD and SU + WLD. As seen in Table 4, the LD blocks defined by the suggestive SNPs had PVE between 0.06 and 0.08, depending on the dataset (SU, WLD, SU + WLD, or NICL), all of which were significantly greater than zero (permuted *p* < 0.05). In contrast, the genome-wide chunks with the same length as the LD blocks had an average PVE ∼0.008, with PVE increasingly slightly with increasing chunk size, and average permuted *p* values ∼0.5.

### Random Forest GWAS

The RF models for all four datasets were not significantly more accurate at predicting the phenotype in the test population compared to the non-information rate (i.e., the frequency of the more common phenotype), indicating that the RF models were overfitted (Brieuc et al., 2018) such that the SNPs that passed the significance threshold were likely random noise. Because importance values are assigned and the importance threshold defined after fitting the RF model, some SNPs will always pass the importance threshold. Consequently, the value of these important SNPs and the likelihood that the important SNPs are truly trait linked must be gauged using model validation. In this case, the models were invalidated because of their poor phenotype prediction in the test population, indicating that the SNPs classified to be important were unlikely associated with the phenotype.

Additionally, the genomic regions identified by SNPs that passed the importance threshold did not overlap across the four datasets, despite their shared etiology, or with the genomic regions on BTA8 and BTA13 detected in the GLMM association analyses. Model overfitting combined with the lack of common genomic regions across the four datasets indicated that the RFs were unable to overcome the complex genetic architecture of noninfectious claw lesions and identify genomic regions of biological importance. Thus, downstream analyses to estimate SNP effects and conduct pathway and gene ontology analyses were not pursued.

## Discussion

Using GLMM regression, CBAT, and a RF approach to compare the SNP genotypes of sound controls and various types of noninfectious claw lesion cases, we identified genomic regions associated with susceptibility to these claw lesions. Given the overlapping etiology of the noninfectious claw lesion in this study, we expected that association testing would detect the genomic regions shared across some or all four datasets. Common genomic regions were identified from the GLMM and CBAT approaches, but not for the RF approach. Although RFs are a promising tool to identify loci associated with complex traits, the RF models in this study were overfitted, precluding meaningful interpretation of the SNPs that passed the importance threshold. For GLMM testing and CBAT, the associated region detected on BTA8 for SU appeared to be specific for SU because the analyses for the other claw lesions did not detect this region; a SNP in this region (ARS-BFGL-NGS-108587) has previously been associated with SU (van der Spek et al., 2015b). The SNP detected on BTA13 for WLD increased in significance as cows with SU and other noninfectious lesions were added to the GLMM GWAS and CBAT analysis, implying that these lesions shared a genetic component that was less prevalent in SU cases. LD blocks defined by the top SNPs from the GLMM GWAS with nonzero effects from Bayesian estimation were explored further for candidate genes and previously defined QTL that were also functionally relevant to NICL etiology. The identification of promising candidate genes within the associated regions may lend more confidence to those regions; however, genetic selection does not require candidate gene identification and instead uses markers that are associated with, but not necessarily causal for, the trait. Thus, the candidate genes are presented below to postulate their contribution to etiology rather than to inform genetic selection.

Sole ulcers and WLD are thought to result from increased laxity of the suspensory system from collagen breakdown and a thinner digital cushion, allowing the distal phalanx to rotate and sink within the claw (Lischer et al., 2002; Bicalho et al., 2009; Newsome et al., 2017a,b; Shearer and van Amstel, 2017; Stambuk et al., 2019). As the distal phalanx crushes the underlying corium, a hemorrhage develops at the pressure site and horn production through keratinization in the corium is disrupted, leading to horn thinning and, eventually, a hole in the horn through which the corium protrudes and develops into a SU (Greenough, 2007; Shearer et al., 2015). Similarly, WLD is thought to develop as a result of improper weight bearing and/or flooring causing defective horn production along the white line that is more prone to debris and bacteria infiltration, and when the bacteria reach the corium, an abscess forms (Shearer and van Amstel, 2017). It has been theorized that subclinical laminitis weakens the suspensory system and thereby predisposes the cow to SU and WLD (Thoefner et al., 2004), although evidence supporting this theory is limited (Danscher et al., 2010). New bone development on the third phalanx (Rusterholz, 1920; Blowey et al., 2000; Lischer et al., 2002) is associated with increasing age (Tsuka et al., 2012; Newsome et al., 2016) and is thought to contribute to a higher incidence of ulceration (Rusterholz, 1920; Tsuka et al., 2012). Because foot and leg conformation influences weight distribution within and between claws, the foot and leg conformation traits are thought to be correlated with SU + WLD susceptibility, although stronger evidence is needed to support the low to moderate phenotypic (Capion et al., 2008; Pérez-Cabal and Charfeddine, 2016) and genetic (Chapinal et al., 2013) correlations that were previously observed. Based on the etiology of noninfectious claw lesions and the possible genetic correlation of the susceptibility of these claw lesions with the conformation traits, genes and QTL related to collagen, keratinization, bone growth, adipose, and foot and leg conformation were considered functionally relevant.

For SU, the suggestive SNPs fell in or near *DCAF12* (DDB1 and CUL4-associated factor 12), an evolutionarily conserved apoptosis regulation gene involved in DNA repair and protein degradation that is required for tissue homeostasis under stress conditions, as demonstrated in *Drosophila* (Hwangbo et al., 2016). The metabolic stress associated with NICL could potentially disrupt the regulation of *DCAF12* and contribute to aberrant tissue homeostasis within the claw. Within the LD block, *APTX*, *AQP7*, *B4GALT1*, *ENHO*, *GALT*, *GULO*, and *UBAP2* had functions involved in wound healing, skin lesions, bone growth and mineralization, adipose tissue, and keratin summarized in Table 5. Notably, the LD block included a SNP that van der Spek et al. (2015b) had previously associated with SU susceptibility, ARS-BFGL-NGS-108587, supporting this SNP as a susceptibility locus for SU and the investigation into the region. No other previously defined QTL, physiologically relevant, or foot and leg conformation QTL were identified in the LD block. The two suggestive chunks on BTA17 both fell in *TMEM132B* (transmembrane protein 132B; Table 5), which, in humans, encodes a member of the TMEM132 family of evolutionarily ancient cell adhesion molecules that connect the extracellular medium with the intracellular skeleton (Sanchez-Pulido and Ponting, 2018).

**TABLE 5:** Candidate genes in linkage disequilibrium blocks defined by suggestive SNPs from the generalized linear mixed model and chunk-based association testing for sole ulcers (SU), white line disease (WLD), sole ulcers and/or white line disease (SU** +** WLD), and noninfectious claw lesions (NICL) and the tissues in which they were expressed.

Claw lesion	Gene symbol	Gene description	Functional relevance	RNA tissue specificity
SU	*DCAF12*	DDB1 (damage-specific binding protein) and CUL4 (cullin 4)-associated factor 12	Regulates apoptosis required for tissue homeostasis under stress conditions (Hwangbo et al., 2016)	Ubiquitous
	*APTX*	Aprataxin	Decreased bone mineral content (MGI)	Ubiquitous
			Increased total body fat amount (MGI)	
	*AQP7*	Aquaporin 7	Abnormal white adipose tissue physiology (MGI)	Adipose, cardiovascular, and bone marrow
			Increased fat cell size (MGI)	
	B4GALT1	Beta-1,4-galactosyltransferase 1	Decreased subcutaneous adipose tissue amount (MGI)	Mammary gland
			Delayed wound healing (MGI)	
			Hyperkeratosis (MGI)	
			Skin lesions (MGI)	
			Thin skin (MGI)	
	ENHO	Energy homeostasis associated	Increased body fat mass (MGI)	Central nervous system
			Increased percent body fat/body weight (MGI)	
	GALT	Galactose-1-phosphate uridylyltransferase	Decreased subcutaneous adipose tissue amount (MGI)	Ubiquitous
			Delayed wound healing (MGI)	
			Hyperkeratosis (MGI)	
			Skin lesions (MGI)	
			Thin skin (MGI)	
	GULO	Gulonolactone (L-)oxidase	Abnormal bone mineralization (MGI)	Liver
			Abnormal long bone epiphyseal plate morphology (MGI)	
			Abnormal trabecular bone morphology (MGI)	
			Decreased bone mineral density (MGI)	
			Decreased compact bone thickness (MGI)	
	TMEM132B	Transmembrane protein 132B	Cell adhesion molecule that connects the extracellular medium with the intracellular skeleton (Sanchez-Pulido and Ponting, 2018)	Central nervous system, testes
	UBAP2	Ubiquitin-associated protein 2	Abnormal adipose tissue amount (MGI)	Ubiquitous
WLD, SU + WLD, NICL	DIP2C	Disco-interacting protein 2 homolog C	Regulates DNA methylation and the epithelial–mesenchymal transition in human cell lines (Larsson et al., 2017)	Ubiquitous
			Mutations associated with skeletal dysplasia (Maddirevula et al., 2018)	
	PCNA	Proliferating cell nuclear antigen	Abnormal adipose tissue development (MGI)	Ubiquitous
			Decreased percent body fat/body weight (MGI)	
			Decreased white fat cell number	
	RASSF2	Ras association (RalGDS/AF-6) domain family member 2	Abnormal bone mineralization (MGI)	White blood cells and immune tissues
			Abnormal trabecular bone morphology (MGI)	
			Decreased bone marrow cell number (MGI)	
			Decreased bone mass (MGI)	
			Decreased bone mineral density (MGI)	
			Decreased bone trabecula number (MGI)	
			Decreased trabecular bone thickness (MGI)	
			Decreased trabecular bone volume (MGI)	
	WDR37	WD repeat domain 37	Increased bone mineral content (MGI)	Ubiquitous

*MGI, mammalian phenotype associated with gene from the Mammalian Genome Informatics batch query.*

For NICL, all nine suggestive SNPs fell directly upstream or within introns of *DIP2C* (disco-interacting protein 2 homolog C), which is hypothesized to play a role in transcription and methylation regulation. *DIP2C* has been shown to regulate DNA methylation and the epithelial–mesenchymal transition in human cell lines (Larsson et al., 2017), and mutations in *DIP2C* have been associated with skeletal dysplasia affecting bone and cartilage development in humans (Maddirevula et al., 2018). The LD block contained three additional candidate genes with functions related to adipose tissue, bone growth, and bone mineralization (Table 5). The LD block on BTA13 did not overlap with previously defined QTL that were apparently related to NICL or foot and leg conformation traits. According to the Cattle Gene Atlas (Fang et al., 2020), some candidate genes were expressed ubiquitously (*DCAF12*, *APTX*, *GALT*, *UBAP2*, *DIP2C*, *PCNA*, and *WDR37*), and others were expressed more highly in specific tissues, such as adipose, cardiovascular, bone marrow, central nervous system, mammary, liver, or immune tissues (*AQP7*, *B4GALT1*, *ENHO*, *GULO*, and *RASSF2*; Table 5).

Prior GWAS studies of NICL, while having larger sample sizes, were sampled from larger geographical regions and used lower-density SNP panels. An acknowledged limitation of this study is the small sample size. However, previous GWAS with smaller sample sizes using the high-density SNP array were able to detect associated loci in Holstein populations for digital cushion thickness (*n* = 502) (Stambuk et al., 2020) and left displaced abomasum (*n* = 406) (Lehner et al., 2018), implying that locus detection is possible despite smaller sample sizes. By maintaining stringent phenotyping for sound controls, minimizing environmental and housing variability, and increasing SNP density, we aimed to optimize the ability to detect genomic variants at the expense of larger sample sizes. Additionally, the CBAT approach reduced the number of tests performed to increase power and found the same regions of association, providing further support for these regions. Because SU susceptibility is also affected by environmental management, including housing and nutrition, we sought to minimize environmental variability by sampling cows at dairies with similar nutrition and flooring, as the diets fed at the five dairies were similar and all dairies used a freestall flush barn system and rubber flooring in alleys.

Whereas previous published studies of noninfectious claw lesions have not used the high-density panel, our study with the 777K SNP panel allowed for higher resolution when defining the LD blocks. Furthermore, RF analysis and Bayesian regression methods were implemented to perform joint association testing of multiple top SNPs while working around the uneven sampling of controls. The two published GWAS for SU susceptibility found associated SNPs on different chromosomes than those identified in this study, specifically on BTA 8, 10, 11, 18, and 22 using a linear animal model (van der Spek et al., 2015b) and on BTA12 and 25 using a linear mixed model (Sánchez-Molano et al., 2019). Other GWAS for traits related to SU + WLD included digital cushion thickness (Sánchez-Molano et al., 2019; Stambuk et al., 2020), sole hemorrhage susceptibility (van der Spek et al., 2015b; Sánchez-Molano et al., 2019), and laminitis susceptibility (Naderi et al., 2018), although the SNPs detected in these studies were also on different chromosomes from those from this study.

Because noninfectious claw lesions have a similar etiology, it has been postulated that pleiotropy may exist across the different noninfectious claw lesions and related traits. For instance, estimates of the genetic correlation between SU and WLD are significant, ranging from 0.41 to 0.60 depending on parity (van der Linde et al., 2010). However, past GWAS have not found associations on the same chromosomes among SU, WLD, digital cushion thickness, sole hemorrhage, or laminitis (van der Spek et al., 2015b; Naderi et al., 2018; Sánchez-Molano et al., 2019), or if SNPs from the same chromosome were detected, they were in different regions. Specifically, the only common chromosome among these three GWAS was BTA11: van der Spek et al. (2015b) found Hapmap38795-BTA-97039 for SU at BTA11:23302850, and Naderi et al. (2018) found BTB-00466773 for laminitis at BTA11:48309332 (the SNP positions were updated to the ARS-UCD1.2 map). The QTL identified on BTA13 may thus represent a portion of the common genetic contribution to the different types of noninfectious claw lesions.

## Conclusion

Using logistic mixed model single-marker regression and CBAT, genomic regions associated with susceptibility were identified on BTA8 for SU and BTA13 for WLD, SU + WLD, and NICL. The associated regions on BTA8 and BTA13 contained candidate genes related to wound healing, skin lesions, bone growth and mineralization, adipose tissue, and keratin. The RF approach was unable to overcome the complexity of these lesion traits and reliably identify potential candidate QTL. Although these findings must be validated in larger populations in other geographical regions, the detection of a region associated with SU susceptibility that included a previously reported locus suggested that the study cohort was adequate to identify the regions of susceptibility for NICL. Further exploration of these regions through targeted sequencing or RNA-seq in claw tissues with and without noninfectious claw lesions may uncover variants in the genes or regulatory elements contributing to lameness. The multiplicity of associations detected in this and other studies demonstrated the complexity of the genetic architecture underlying noninfectious claw lesion susceptibility.

## Data Availability Statement

The microarray datasets generated for this study can be found in NCBI’s Gene Expression Omnibus data repository (GEO series record GSE159157 and GSE165945).

## Ethics Statement

The animal study was reviewed and approved by the Institutional Animal Care and Use Committee. Written informed consent was obtained from the owners for the participation of their animals in this study.

## Author Contributions

AO, TF, AD, and EL conceptualized the research aims with supervision from AO and TF and prepared and edited the manuscript. AD and EL arranged the blood sample collection and processing, curated hoof trimming records, developed the methodology, and performed the computational analyses. TF was instrumental in developing code for the computational analyses. AO provided the funding, lab space, and computing resources for this research. All authors contributed to the article and approved the submitted version.

## Conflict of Interest

The authors declare that the research was conducted in the absence of any commercial or financial relationships that could be construed as a potential conflict of interest.

## Abbreviations

BTA: *Bos taurus* autosome
CBAT: chunk-based association testing
GLMM: generalized linear mixed model
GRM: genetic relatedness matrix
GWAS: genome-wide association studies
LD: linkage disequilibrium
MAF: minor allele frequency
NICL: noninfectious claw lesions
PVE: proportion of phenotypic variance explained
RF: random forest
SUs: sole ulcers
WLD: white line disease.

## References

[B1] Bicalho R. C. Machado V. S. Caixeta L. S. (2009). Lameness in dairy cattle: A debilitating disease or a disease of debilitated cattle? A cross-sectional study of lameness prevalence and thickness of the digital cushion. *J. Dairy Sci.* 92 3175–3184. 10.3168/jds.2008-1827 19757545

[B2] Blowey R. W. Ossent P. Watson C. L. Hedges V. Green L. E. Packington A. J. (2000). Possible distinction between sole ulcers and heel ulcers as a cause of bovine lameness. *Vet. Rec.* 147 110–112. 10.1136/vr.147.4.110 10955883

[B3] Brieuc M. S. O. Waters C. D. Drinan D. P. Naish K. A. (2018). A practical introduction to random forest for genetic association studies in ecology and evolution. *Mol. Ecol. Resour.* 18 755–766. 29504715 10.1111/1755-0998.12773

[B4] Browning B. L. Zhou Y. Browning S. R. (2018). A one-penny imputed genome from next-generation reference panels. *Am. J. Hum. Genet.* 103 338–348. 10.1016/j.ajhg.2018.07.015 30100085 PMC6128308

[B5] Capion N. Thamsborg S. M. Enevoldsen C. (2008). Conformation of hind legs and lameness in danish holstein heifers. *J. Dairy Sci.* 91 2089–2097. 10.3168/jds.2006-457 18420640

[B6] Cha E. Hertl J. A. Bar D. Gröhn Y. T. Grohn Y. T. (2010). The cost of different types of lameness in dairy cows calculated by dynamic programming. *Prev. Vet. Med. J.* 97 1–8. 10.1016/j.prevetmed.2010.07.011 20801533

[B7] Chang C. C. Chow C. C. Tellier L. C. Vattikuti S. Purcell S. M. Lee J. J. (2015). Second-generation PLINK: rising to the challenge of larger and richer datasets. *Gigascience* 4:7. 10.1186/s13742-015-0047-8 25722852 PMC4342193

[B8] Chapinal N. Koeck A. Sewalem A. Kelton D. F. Mason S. Cramer G. (2013). Genetic parameters for hoof lesions and their relationship with feet and leg traits in Canadian Holstein cows. *J. Dairy Sci.* 96 2596–2604. 10.3168/jds.2012-6071 23415531

[B9] Charfeddine N. Pérez-Cabal M. A. (2017). Effect of claw disorders on milk production, fertility, and longevity, and their economic impact in Spanish Holstein cows. *J. Dairy Sci.* 100 653–665. 10.3168/jds.2016-11434 27865503

[B10] Chen H. Wang C. Conomos M. P. Stilp A. M. Li Z. Sofer T. (2016). Control for population structure and relatedness for binary traits in genetic association studies via logistic mixed models. *Am. J. Hum. Genet.* 98 653–666. 10.1016/j.ajhg.2016.02.012 27018471 PMC4833218

[B11] Christopoulos D. (2017). Introducing unit invariant knee (UIK) as an objective choice for elbow point in multivariate data analysis techniques. *SSRN Electron. J.* 10.2139/ssrn.3043076

[B12] Christopoulos D. T. (2016). On the efficient identification of an inflection point on the efficient identification of an inflection point. *Int. J. Math. Sci. Comput.* 6 13–20.

[B13] Cramer G. Lissemore K. D. Guard C. L. Leslie K. E. Kelton D. F. (2008). Herd- and cow-level prevalence of foot lesions in ontario dairy cattle. *J. Dairy Sci.* 91 3888–3895. 10.3168/jds.2008-1135 18832211

[B14] Danscher A. M. Toelboell T. H. Wattle O. (2010). Biomechanics and histology of bovine claw suspensory tissue in early acute laminitis. *J. Dairy Sci.* 93 53–62. 10.3168/jds.2009-2038 20059904

[B15] DeFrain J. M. Socha M. T. Tomlinson D. J. (2013). Analysis of foot health records from 17 confinement dairies. *J. Dairy Sci.* 96 7329–7339. 10.3168/jds.2012-6017 23992979

[B16] Dolecheck K. A. Overton M. W. Mark T. B. Bewley J. M. (2019). Use of a stochastic simulation model to estimate the cost per case of digital dermatitis, sole ulcer, and white line disease by parity group and incidence timing. *J. Dairy Sci.* 102 715–730. 10.3168/jds.2018-14901 30415843

[B17] Eicher S. D. Lay D. C. Arthington J. D. Schutz M. M. (2013). Effects of rubber flooring during the first 2 lactations on production, locomotion, hoof health, immune functions, and stress1. *J. Dairy Sci.* 96 3639–3651. 10.3168/jds.2012-6049 23587383

[B18] Fang L. Cai W. Liu S. Canela-Xandri O. Gao Y. Jiang J. (2020). Comprehensive analyses of 723 transcriptomes enhance genetic and biological interpretations for complex traits in cattle. *Genome Res.* 30 790–801. 10.1101/gr.250704.119 32424068 PMC7263193

[B19] Fjeldaas T. Sogstad ÅM. Østerås O. (2011). Locomotion and claw disorders in Norwegian dairy cows housed in freestalls with slatted concrete, solid concrete, or solid rubber flooring in the alleys. *J. Dairy Sci.* 94 1243–1255. 10.3168/jds.2010-3173 21338790

[B20] Functional Annotation of Animal Genomes (FAANG) (2019). *FAANG Mine.* Available online at: http://128.206.116.18:8080/faangmine/begin.do (accessed August 3, 2020)

[B21] Gabry J. Simpson D. Vehtari A. Betancourt M. Gelman A. (2019). Visualization in bayesian workflow. *J. R. Stat. Soc. Ser. A* 182 389–402. 10.1111/rssa.12378

[B22] Gelman A. Su Y.-S. Yajima M. Hill J. Pittau M. G. Kerman J. (2020). *R Package ARM: Data Analysis Using Regression and Multilevel/Hierarchical Models.* Available online at: https://cran.r-project.org/package=arm (accessed January 21, 2021).

[B23] Goldstein B. A. Hubbard A. E. Cutler A. Barcellos L. F. (2010). An application of random forests to a genome-wide association dataset: methodological considerations and new findings. *BMC Genet.* 11:49. 10.1186/1471-2156-11-49 20546594 PMC2896336

[B24] Goodrich B. Gabry J. Ali I. Brilleman S. (2020). *Rstanarm: Bayesian Applied Regression Modeling Via Stan.* Available online at: https://mc-stan.org/rstanarm/ (accessed January 21, 2021).

[B25] Green L. E. Borkert J. Monti G. Tadich N. (2010). Associations between lesion-specific lameness and the milk yield of 1,635 dairy cows from seven herds in the Xth region of Chile and implications for management of lame dairy cows worldwide. *Anim. Welf.* 19 419–427.

[B26] Green L. E. Hedges V. J. Schukken Y. H. Blowey R. W. Packington A. J. (2002). The impact of clinical lameness on the milk yield of dairy cows. *J. Dairy Sci.* 85 2250–2256. 12362457 10.3168/jds.S0022-0302(02)74304-X

[B27] Greenough P. R. (2007). *Bovine Laminitis and Lameness*, 1st Edn, eds Bergsten C. Brizzir A. Mülling C. K. W. (Canada: Elsevier Ltd), 10.1016/B978-0-7020-2780-2.X5001-0

[B28] Häggman J. Juga J. (2013). Genetic parameters for hoof disorders and feet and leg conformation traits in Finnish Holstein cows. *J. Dairy Sci.* 96 3319–3325. 10.3168/jds.2012-6334 23498009

[B29] Häggman J. Juga J. Sillanpää M. J. Thompson R. (2013). Genetic parameters for claw health and feet and leg conformation traits in Finnish Ayrshire cows. *J. Anim. Breed. Genet.* 130 89–97. 10.1111/j.1439-0388.2012.01007.x 23496009

[B30] Hernandez J. A. Garbarino E. J. Shearer J. K. Risco C. A. Thatcher W. W. (2005). Comparison of milk yield in dairy cows with different degrees of lameness. *J. Am. Vet. Med. Assoc.* 227 1292–1296. 10.2460/javma.2005.227.1292 16266019

[B31] Hu Z. L. Park C. A. Reecy J. M. (2019). Building a livestock genetic and genomic information knowledgebase through integrative developments of Animal QTLdb and CorrDB. *Nucleic Acids Res.* 47 D701–D710. 10.1093/nar/gky1084 30407520 PMC6323967

[B32] Hwangbo D. S. Biteau B. Rath S. Kim J. Jasper H. (2016). Control of apoptosis by Drosophila DCAF12. *Dev. Biol.* 413 50–59. 10.1016/j.ydbio.2016.03.003 26972874 PMC5106244

[B33] Koufariotis L. Chen Y. P. P. Bolormaa S. Hayes B. J. (2014). Regulatory and coding genome regions are enriched for trait associated variants in dairy and beef cattle. *BMC Genomics* 15:1–16. 10.1186/1471-2164-15-436 24903263 PMC4070550

[B34] Koufariotis L. T. Chen Y. P. P. Stothard P. Hayes B. J. (2018). Variance explained by whole genome sequence variants in coding and regulatory genome annotations for six dairy traits. *BMC Genomics* 19:237. 10.1186/s12864-018-4617-x 29618315 PMC5885354

[B35] Kuhn M. (2008). Building predictive models in R using the caret package. *J. Stat. Softw.* 28 1–26. 10.18637/jss.v028.i0527774042

[B36] Lai E. Danner A. L. Famula T. R. Oberbauer A. M. (2020). Genome-wide association studies reveal susceptibility loci for digital dermatitis in holstein cattle. *Animals* 10:2009. 10.3390/ani10112009 33142934 PMC7693332

[B37] Lander E. Kruglyak L. (1995). Genetic dissection of complex traits: Guidelines for interpreting and reporting linkage results. *Nat. Genet.* 11 241–247. 10.1038/ng1195-241 7581446

[B38] Larsson C. Ali M. A. Pandzic T. Lindroth A. M. He L. Sjöblom T. (2017). Loss of DIP2C in RKO cells stimulates changes in DNA methylation and epithelial-mesenchymal transition. *BMC Cancer* 17:487. 10.1186/s12885-017-3472-5 28716088 PMC5513093

[B39] Lehner S. Zerbin I. Doll K. Rehage J. Distl O. (2018). A genome-wide association study for left-sided displacement of the abomasum using a high-density single nucleotide polymorphism array. *J. Dairy Sci.* 101 1258–1266. 10.3168/jds.2017-13216 29224884

[B40] Li M.-X. Yeung J. M. Y. Cherny S. S. Sham P. C. (2012). Evaluating the effective numbers of independent tests and significant p-value thresholds in commercial genotyping arrays and public imputation reference datasets. *Hum. Genet.* 131 747–756. 10.1007/s00439-011-1118-2 22143225 PMC3325408

[B41] Lischer C. J. Ossent P. Räber M. Geyer H. (2002). Suspensory structures and supporting tissues of the third phalanx of cows and their relevance to the development of typical sole ulcers (*Rusterholz ulcers*). *Vet. Rec.* 151 694–698. 10.1136/vr.151.23.694 12503788

[B42] Listgarten J. Lippert C. Kang E. Y. Xiang J. Kadie C. M. Heckerman D. (2013). A powerful and efficient set test for genetic markers that handles confounders. *Bioinformatics* 29 1526–1533. 10.1093/bioinformatics/btt177 23599503 PMC3673214

[B43] Maddirevula S. Alsahli S. Alhabeeb L. Patel N. Alzahrani F. Shamseldin H. E. (2018). Expanding the phenome and variome of skeletal dysplasia. *Genet. Med.* 20 1609–1616. 10.1038/gim.2018.50 29620724

[B44] Makanjuola B. O. Miglior F. Abdalla E. A. Maltecca C. Schenkel F. S. Baes C. F. (2020). Effect of genomic selection on rate of inbreeding and coancestry and effective population size of Holstein and Jersey cattle populations. *J. Dairy Sci.* 103 5183–5199. 10.3168/jds.2019-18013 32278553

[B45] Malchiodi F. Koeck A. Chapinal N. Sargolzaei M. Fleming A. Kelton D. F. (2015a). Genetic analyses of hoof lesions in canadian holsteins using an alternative contemporary group. *Int. Bull.* 49 64–68.

[B46] Malchiodi F. Koeck A. Christen A. M. Schenkel F. S. Kelton D. F. Miglior F. (2015b). *Genetic Parameters and Genome Wide Association Study of Individual Hoof Lesions in Canadian Holsteins Using Different Contemporary Groups.* Guelph: Canadian Dairy Network.

[B47] Moore C. M. Jacobson S. A. Fingerlin T. E. (2019). Power and sample size calculations for genetic association studies in the presence of genetic model misspecification. *Hum. Hered.* 84 256–271. 10.1159/000508558 32721961 PMC7666027

[B48] Mostert P. F. van Middelaar C. E. de Boer I. J. M. Bokkers E. A. M. (2018). The impact of foot lesions in dairy cows on greenhouse gas emissions of milk production. *Agric. Syst.* 167 206–212. 10.1016/j.agsy.2018.09.006

[B49] Naderi S. Bohlouli M. Yin T. Konig S. König S. (2018). Genomic breeding values, SNP effects and gene identification for disease traits in cow training sets. *Anim. Genet.* 49 178–192. 10.1111/age.12661 29624705

[B50] National Research Council (NRC) (2001). *Nutrient Requirements of Dairy Cattle*, 7th Edn. Washington, D.C.: National Academies Press, 10.17226/9825

[B51] Newsome R. F. Green M. J. Bell N. J. Bollard N. J. Mason C. S. Whay H. R. (2017a). A prospective cohort study of digital cushion and corium thickness. part 1: associations with body condition, lesion incidence, and proximity to calving. *J. Dairy Sci.* 100 4745–4758. 10.3168/jds.2016-12012 28434744

[B52] Newsome R. F. Green M. J. Bell N. J. Bollard N. J. Mason C. S. Whay H. R. (2017b). A prospective cohort study of digital cushion and corium thickness. part 2: does thinning of the digital cushion and corium lead to lameness and claw horn disruption lesions? *J. Dairy Sci.* 100 4759–4771. 10.3168/jds.2016-12013 28434731

[B53] Newsome R. F. Green M. J. Bell N. J. Chagunda M. G. G. Mason C. S. Rutland C. S. (2016). Linking bone development on the caudal aspect of the distal phalanx with lameness during life. *J. Dairy Sci.* 99 4512–4525. 10.3168/jds.2015-10202 27060810

[B54] Oberbauer A. M. Berry S. L. Belanger J. M. McGoldrick R. M. Pinos-Rodriquez J. M. Famula T. R. (2013). Determining the heritable component of dairy cattle foot lesions. *J. Dairy Sci.* 96 605–613. 10.3168/jds.2012-5485 23063151

[B55] Pérez-Cabal M. A. Charfeddine N. (2016). Short communication: association of foot and leg conformation and body weight with claw disorders in Spanish Holstein cows. *J. Dairy Sci.* 99 9104–9108. 10.3168/jds.2016-11331 27614841

[B56] Purcell S. M. Chang C. C. (2015). *Plink 1.9.* Available online at: https://www.cog-genomics.org/plink2 (accessed December 4, 2020).

[B57] R Development Core Team (2010). *R: A Language and Environment for Statistical Computing.* Available online at: https://www.r-project.org/ (accessed January 21, 2021).

[B58] Richardson I. W. Berry D. P. Wiencko H. L. Higgins I. M. More S. J. McClure J. (2016). A genome-wide association study for genetic susceptibility to Mycobacterium bovis infection in dairy cattle identifies a susceptibility QTL on chromosome 23. *Genet. Sel. Evol.* 48:19. 10.1186/s12711-016-0197-x 26960806 PMC4784436

[B59] Ring S. C. Twomey A. J. Byrne N. Kelleher M. M. Pabiou T. Doherty M. L. (2018). Genetic selection for hoof health traits and cow mobility scores can accelerate the rate of genetic gain in producer-scored lameness in dairy cows. *J. Dairy Sci.* 101 10034–10047. 10.3168/jds.2018-15009 30219421

[B60] Rosen B. D. Bickhart D. M. Schnabel R. D. Koren S. Elsik C. G. Tseng E. (2020). De novo assembly of the cattle reference genome with single-molecule sequencing. *Gigascience* 9 1–9. 10.1093/gigascience/giaa021 32191811 PMC7081964

[B61] Rusterholz A. (1920). Das spezifisch-traumatische Klauensohlengeschwür des Rindes (the specific traumatic sole ulcer of claws in cattle). *Schweiz. Arch. Tierheilkd.* 62 505–525.

[B62] Sánchez-Molano E. Bay V. Smith R. F. Oikonomou G. Banos G. (2019). Quantitative trait loci mapping for lameness associated phenotypes in holstein–friesian dairy cattle. *Front. Genet.* 10:926. 10.3389/fgene.2019.00926 31636655 PMC6787292

[B63] Sanchez-Pulido L. Ponting C. P. (2018). TMEM132: an ancient architecture of cohesin and immunoglobulin domains define a new family of neural adhesion molecules. *Bioinformatics* 34 721–724. 10.1093/bioinformatics/btx689 29088312 PMC6030884

[B64] Shearer J. K. Plummer P. Schleining J. (2015). Perspectives on the treatment of claw lesions in cattle. *Vet. Med. Res. Reports* 6 273–292. 10.2147/vmrr.s62071 30101113 PMC6067775

[B65] Shearer J. K. van Amstel S. R. (2001). Functional and corrective claw trimming. *Vet. Clin. North Am. - Food Anim. Pract.* 17 53–72. 10.1016/S0749-0720(15)30054-2 11320699

[B66] Shearer J. K. van Amstel S. R. (2017). Pathogenesis and treatment of sole ulcers and white line disease. *Vet. Clin. North Am. Food Anim. Pract.* 33 283–300. 10.1016/j.cvfa.2017.03.001 28442154

[B67] Smith C. L. Eppig J. T. (2009). The mammalian phenotype ontology: enabling robust annotation and comparative analysis. *Wiley Interdiscip. Rev. Syst. Biol. Med.* 1 390–399. 10.1002/wsbm.44 20052305 PMC2801442

[B68] Speed D. Hemani G. Johnson M. R. Balding D. J. (2012). Improved heritability estimation from genome-wide SNPs. *Am. J. Hum. Genet.* 91 1011–1021. 10.1016/j.ajhg.2012.10.010 23217325 PMC3516604

[B69] Stambuk C. R. McArt J. A. A. Bicalho R. C. Miles A. M. Huson H. J. (2019). A longitudinal study of digital cushion thickness and its function as a predictor for compromised locomotion and hoof lesions in Holstein cows. *Transl. Anim. Sci.* 3 74–83. 10.1093/tas/txy107 32704780 PMC7200577

[B70] Stambuk C. R. Staiger E. A. Nazari-Ghadikolaei A. Heins B. J. Huson H. J. (2020). Phenotypic characterization and genome-wide association studies of digital cushion thickness in Holstein cows. *J. Dairy Sci.* 103 3289–3303. 10.3168/jds.2019-17409 32037162

[B71] Thoefner M. B. Pollitt C. C. Van Eps A. W. Milinovich G. J. Trott D. J. Wattle O. (2004). Acute bovine laminitis: a new induction model using alimentary oligofructose overload. *J. Dairy Sci.* 87 2932–2940. 10.3168/jds.S0022-0302(04)73424-4 15375054

[B72] Tsuka T. Ooshita K. Sugiyama A. Osaki T. Okamoto Y. Minami S. (2012). Quantitative evaluation of bone development of the distal phalanx of the cow hind limb using computed tomography. *J. Dairy Sci.* 95 127–138. 10.3168/jds.2011-4316 22192192

[B73] Turner S. D. (2014). qqman: an R package for visualizing GWAS results using QQ and manhattan plots. *bioRxiv [perprint]*:5165. 10.1101/005165

[B74] Twomey A. J. Berry D. P. Evans R. D. Doherty M. L. Graham D. A. Purfield D. C. (2019). Genome-wide association study of endo-parasite phenotypes using imputed whole-genome sequence data in dairy and beef cattle. *Genet. Sel. Evol.* 51 1–17. 10.1186/s12711-019-0457-7 30999842 PMC6471778

[B75] van der Linde C. de Jong G. Koenen E. P. C. Eding H. (2010). Claw health index for Dutch dairy cattle based on claw trimming and conformation data. *J. Dairy Sci.* 93 4883–4891. 10.3168/jds.2010-3183 20855023

[B76] van der Spek D. van Arendonk J. A. M. Bovenhuis H. (2015a). Genetic relationships between claw health traits of dairy cows in different parities, lactation stages, and herds with different claw disorder frequencies. *J. Dairy Sci.* 98 6564–6571. 10.3168/jds.2015-9561 26142850

[B77] van der Spek D. van Arendonk J. A. M. Bovenhuis H. (2015b). Genome-wide association study for claw disorders and trimming status in dairy cattle. *J. Dairy Sci.* 98 1286–1295. 10.3168/jds.2014-8302 25497826

[B78] van der Spek D. van Arendonk J. A. M. Vallée A. A. A. Bovenhuis H. (2013). Genetic parameters for claw disorders and the effect of preselecting cows for trimming. *J. Dairy Sci.* 96 6070–6078. 10.3168/jds.2013-6833 23849633

[B79] Van der Waaij E. H. Holzhauer M. Ellen E. Kamphuis C. De Jong G. (2005). Genetic parameters for claw disorders in Dutch dairy cattle and correlations with conformation traits. *J. Dairy Sci.* 88 3672–3678. 16162542 10.3168/jds.S0022-0302(05)73053-8

[B80] Vanegas J. Overton M. Berry S. L. Sischo W. M. (2006). Effect of rubber flooring on claw health in lactating dairy cows housed in free-stall barns. *J. Dairy Sci.* 89 4251–4258. 10.3168/jds.S0022-0302(06)72471-7 17033012

[B81] Vehtari A. Gabry J. Magnusson M. Yao Y. Andrew Y. Bürkner P.-C. (2020). *Loo: Efficient Leave-One-Out Cross-Validation and WAIC for Bayesian Models.* Available online at: https://mc-stan.org/loo/ (accessed January 21, 2021).

[B82] Vehtari A. Gelman A. Gabry J. (2017). Practical bayesian model evaluation using leave-one-out cross-validation and WAIC. *Stat. Comput.* 27 1413–1432. 10.1007/s11222-016-9696-4

[B83] Xia J. Fan H. Chang T. Xu L. Zhang W. Song Y. (2017). Searching for new loci and candidate genes for economically important traits through gene-based association analysis of Simmental cattle. *Sci. Rep.* 7 1–9. 10.1038/srep42048 28169328 PMC5294460

[B84] Zhou X. Stephens M. (2012). Genome-wide efficient mixed-model analysis for association studies. *Nat. Genet.* 44 821–824. 22706312 10.1038/ng.2310 PMC3386377

