# Supplementary material for: Genome-Wide Association Studies Reveal Susceptibility Loci for Noninfectious Claw Lesions in Holstein Dairy Cattle
Source: Front Genet. 2021 May 28;12:657375. doi: 10.3389/fgene.2021.657375 (PMC8194352; doi:10.3389/fgene.2021.657375)
Supplement: Supplementary file 1 [file Data_Sheet_1.PDF]

Supplementary Material

1 Supplementary Tables

Table S1. Chunks of SNPs that were significant or suggestive in chunk-based association testing and the proportion of phenotypic variance they explained (PVE) for sole ulcers (SU), white line disease (WLD), sole ulcers and/or white line disease (SU+WLD), and noninfectious claw lesions (NICL)

Dataset	BTA	Chunk start (bp)	Chunk end (bp)	Number of SNPs in chunk	PVE (SD)	P	
SU	8	75450001	75550001	13	0.053 (0.044)	1.04E-05	*
	17	50500001	50600001	21	0.113 (0.063)	1.21E-05	*
	17	50550001	50650001	17	0.150 (0.077)	4.76E-06	*
WLD	13	46450001	46550001	5	0.061 (0.058)	1.76E-05	*
SU+WLD	13	46400001	46500001	5	0.093 (0.080)	3.86E-06	*
	13	46450001	46550001	5	0.059 (0.054)	9.73E-07	*
	13	46500001	46600001	7	0.059 (0.051)	2.51E-06	*
	13	46550001	46650001	8	0.061 (0.051)	2.99E-06	*
	13	46600001	46700001	6	0.071 (0.058)	3.18E-06	*
NICL	13	46400001	46500001	5	0.095 (0.081)	2.59E-06	*
	13	46450001	46550001	5	0.060 (0.054)	6.91E-07	**
	13	46500001	46600001	7	0.059 (0.051)	1.83E-06	*
	13	46550001	46650001	8	0.059 (0.049)	2.36E-06	*
	13	46600001	46700001	6	0.069 (0.056)	2.70E-06	*

*PVE by chunk reached genome-wide suggestive significance
**PVE by chunk reached genome-wide significance

2 Supplementary Figures

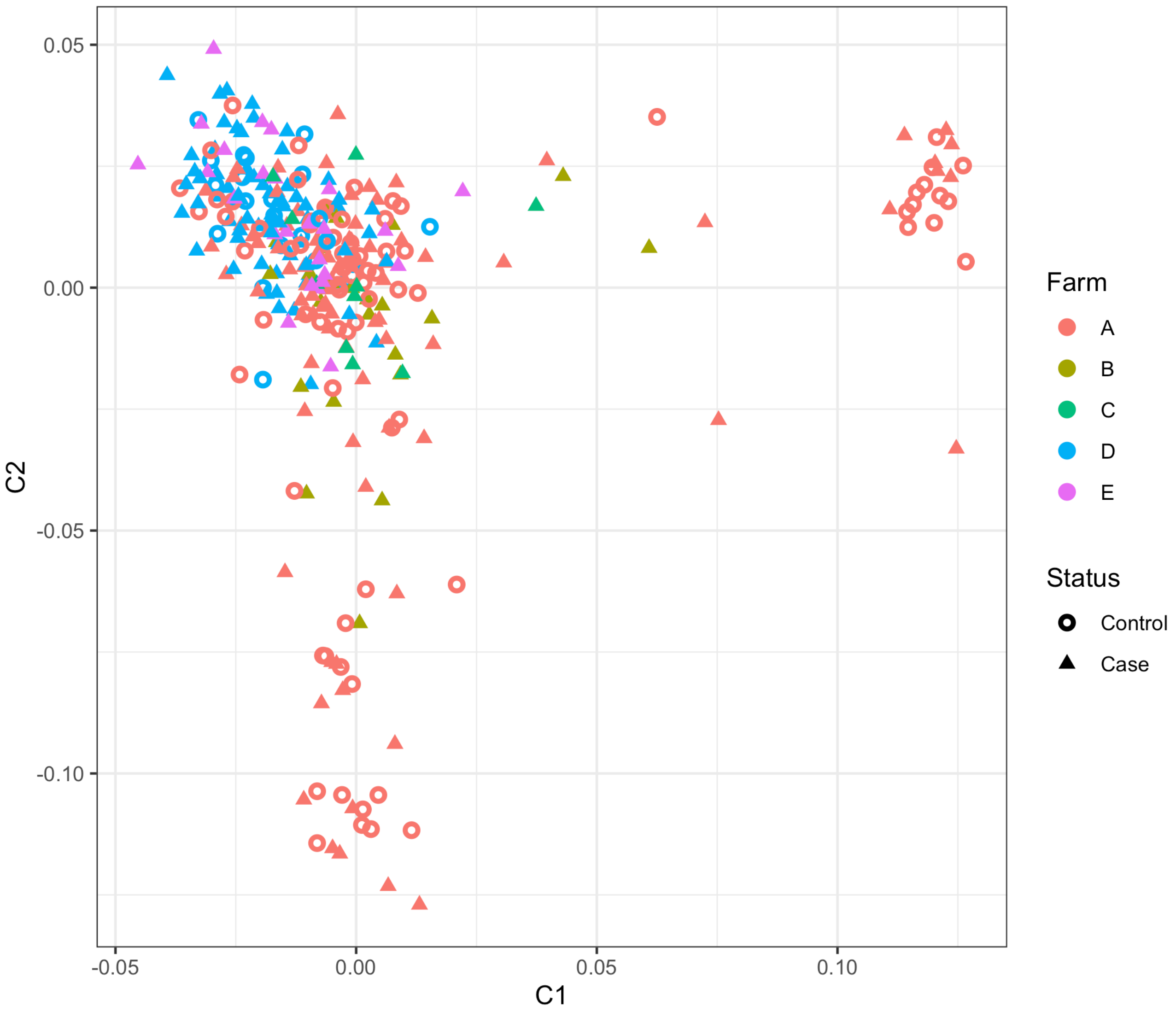

Figure S1. Multidimensional scaling plot showing the first two dimensions for the 217 noninfectious claw lesion cases and 102 sound controls from five dairies used in the genome-wide association analyses.

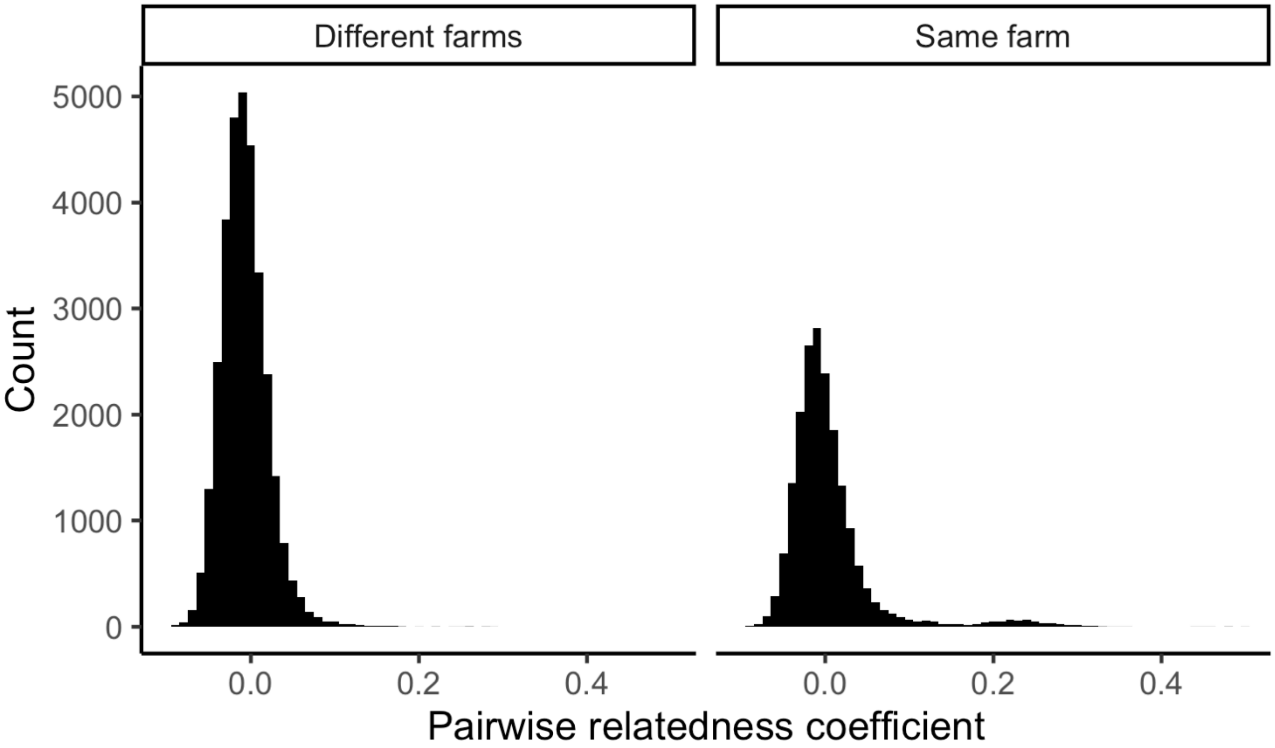

Figure S2. Histogram illustrating the distribution of pairwise relatedness coefficients in cows from the same farm and different farms.

(A)

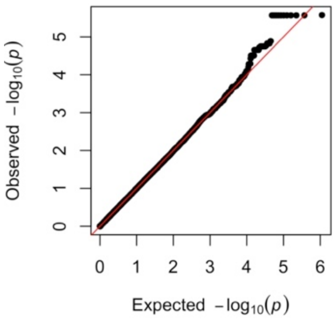

(B)

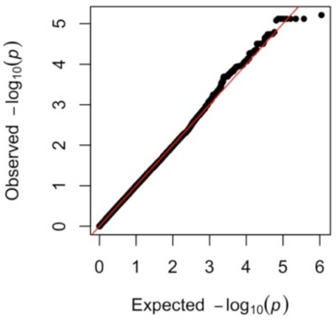

(C)

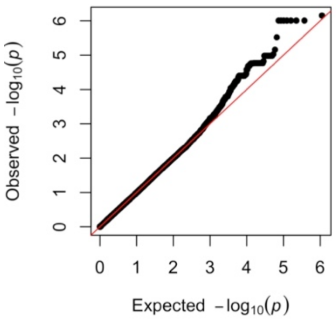

(D)

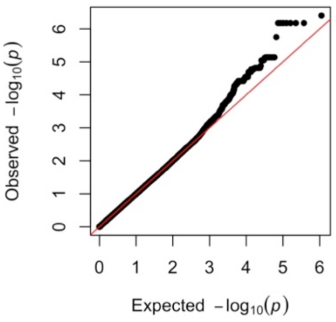

Figure S3. Quantile-quantile plots showing the observed vs. expected p-values outputted from the generalized linear mixed model regression analysis for **(A)** sole ulcers, **(B)** white line disease, **(C)** sole ulcers and white line disease, and **(D)** noninfectious claw lesions. The red line indicates where observed and expected p-values are equivalent.

(A)

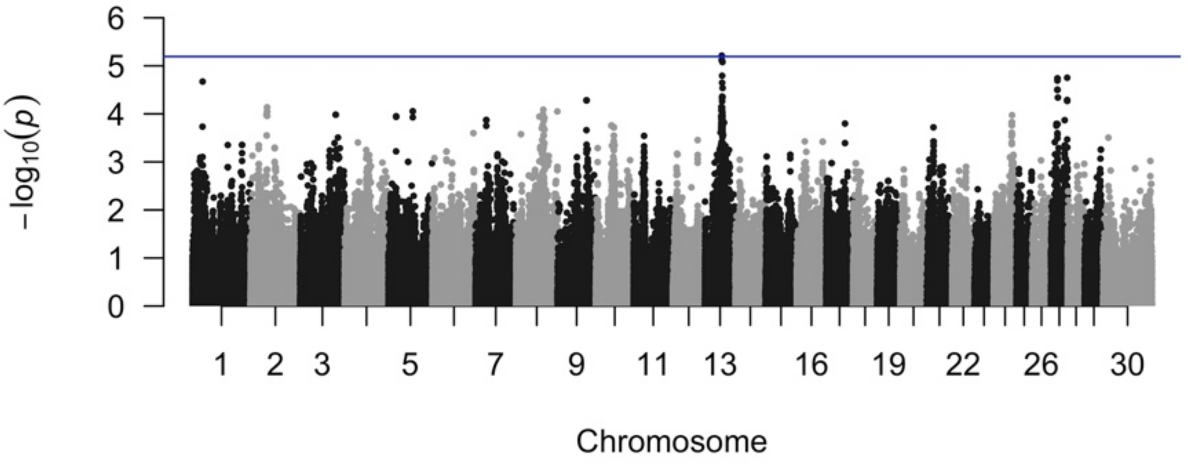

(B)

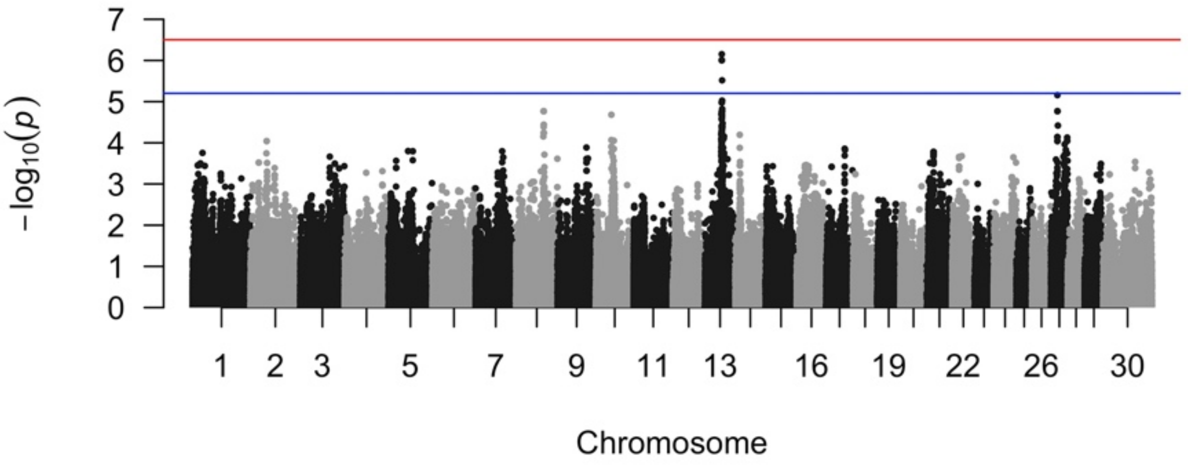

Figure S4. Manhattan plots from the generalized linear mixed model regression association analyses for **(A)** white line disease and **(B)** sole ulcers and white line disease. The blue line indicates the threshold of genome-wide suggestive significance and the red line indicates the threshold of genome-wide significance.

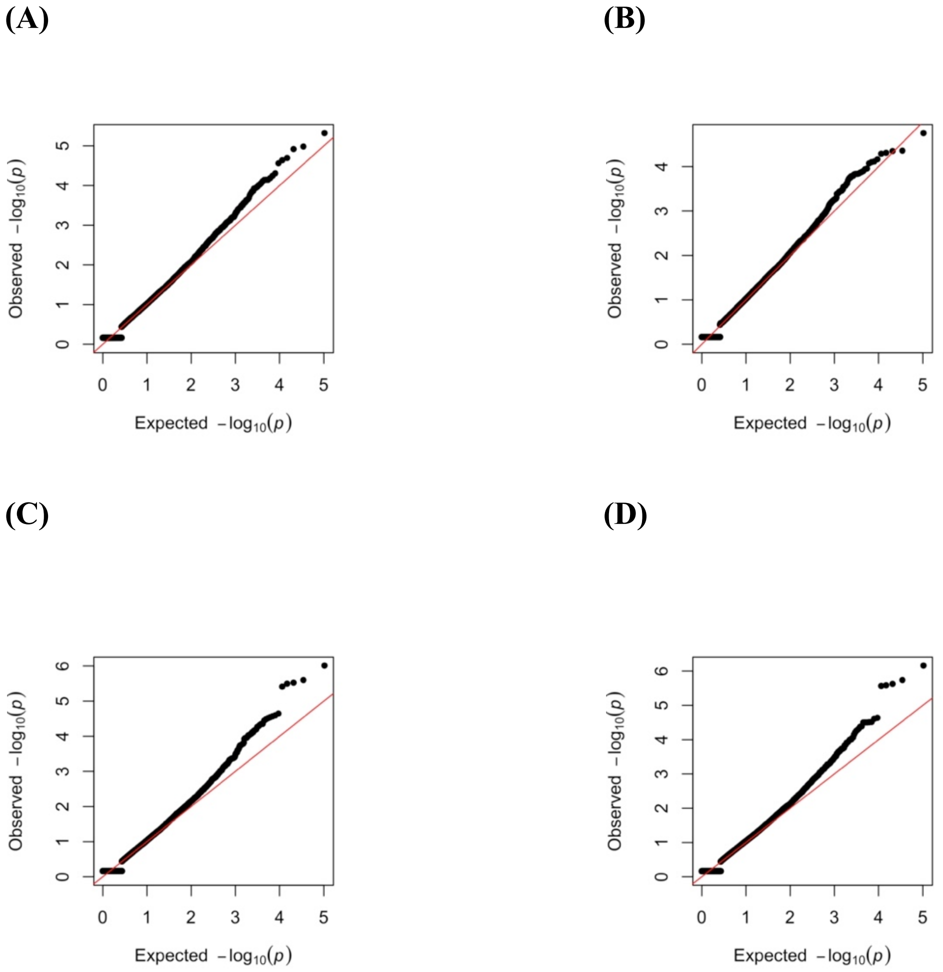

Figure S5. Quantile-quantile plots showing the observed vs. expected p-values outputted from chunk-based association testing for **(A)** sole ulcers, **(B)** white line disease, **(C)** sole ulcers and white line disease, and **(D)** noninfectious claw lesions. The red line indicates where observed and expected p-values are equivalent.

(A)

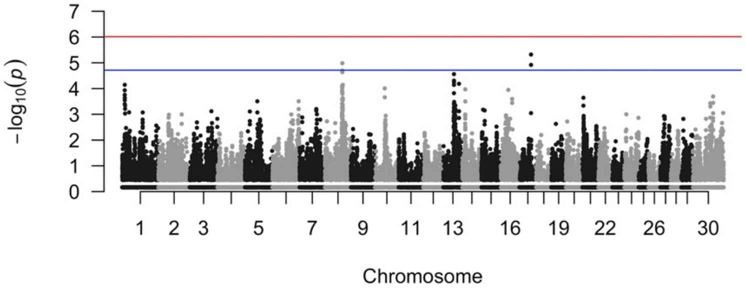

7
6
5
4
3
2
1
0
−log10(p)
1 2 3 5 7 9 11 13 16 19 22 26 30
Chromosome

(B)

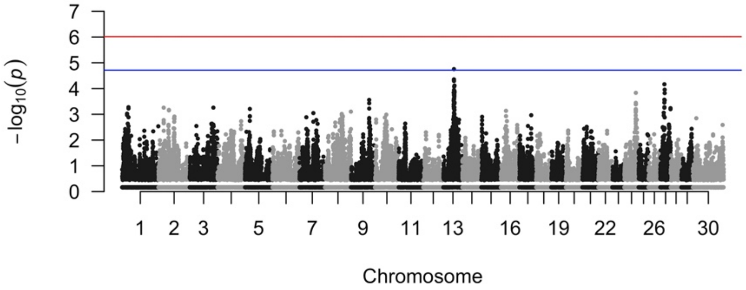

7
6
5
4
3
2
1
0
−log10(p)
1 2 3 5 7 9 11 13 16 19 22 26 30
Chromosome

(C)

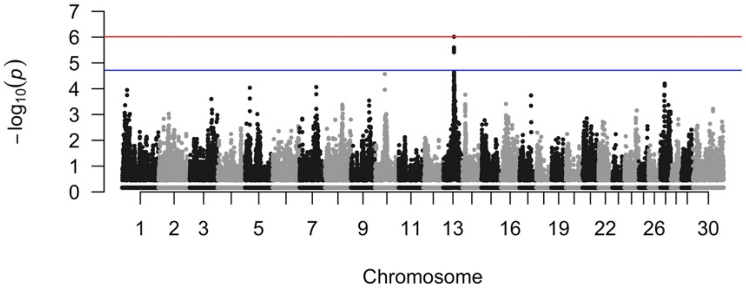

7
6
5
4
3
2
1
0
−log10(p)
1 2 3 5 7 9 11 13 16 19 22 26 30
Chromosome

(D)

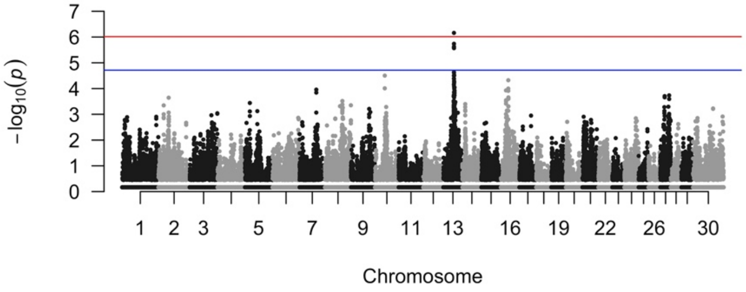

7
6
5
4
3
2
1
0
−log10(p)
1 2 3 5 7 9 11 13 16 19 22 26 30
Chromosome

Figure S6. Manhattan plots from chunk-based association testing using 100 kb chunks spanning the genome for **(A)** sole ulcers, **(B)** white line disease, **(C)** sole ulcers and white line disease, and **(D)** noninfectious claw lesions. The blue line indicates the threshold of genome-wide suggestive significance and the red line indicates the threshold of genome-wide significance.

(A) **(B)**

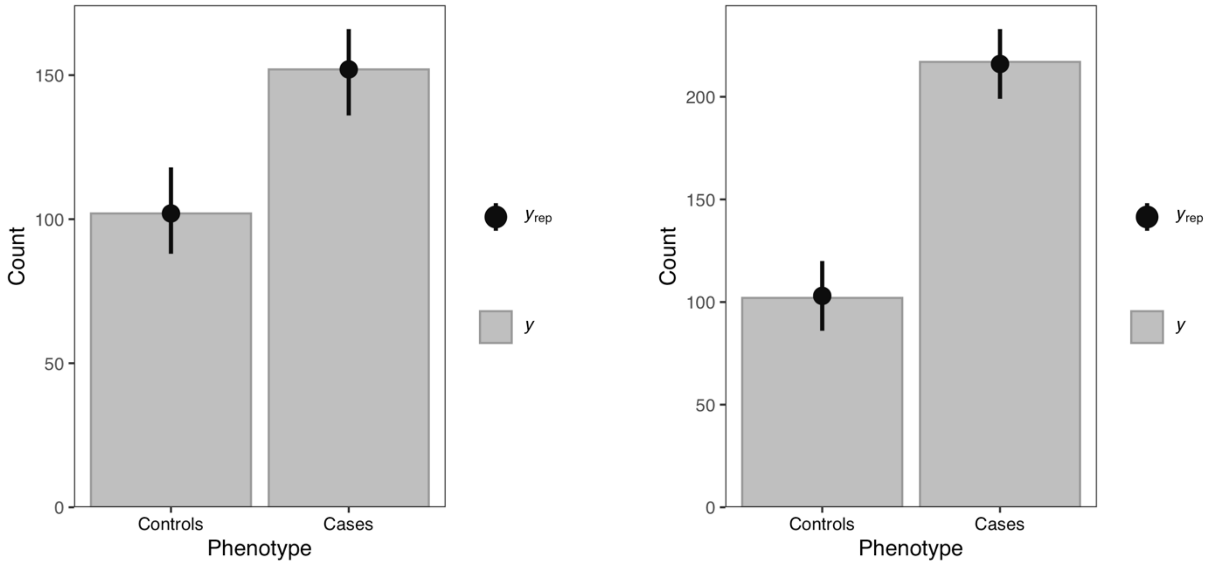

Figure S7. Posterior predictive check for Bayesian estimation of SNP effects of suggestive SNPs from the generalized linear mixed model regression for **(A)** sole ulcers and **(B)** noninfectious claw lesions. Gray bars represent the distribution of observed cases and controls, and black dots with intervals represent the median and uncertainty intervals of replicate phenotypes (y_{rep}) simulated using estimated SNP effects.
